# Exon level machine learning analyses elucidate novel candidate miRNA targets in an avian model of fetal alcohol spectrum disorder

**DOI:** 10.1371/journal.pcbi.1006937

**Published:** 2019-04-11

**Authors:** Abrar E. Al-Shaer, George R. Flentke, Mark E. Berres, Ana Garic, Susan M. Smith

**Affiliations:** 1 Nutrition Research Institute, Department of Nutrition, University of North Carolina at Chapel Hill, Kannapolis, North Carolina, United States of America; 2 Department of Nutritional Sciences, University of Wisconsin-Madison, Madison, Wisconsin, United States of America; National University of Singapore, SINGAPORE

## Abstract

Gestational alcohol exposure causes fetal alcohol spectrum disorder (FASD) and is a prominent cause of neurodevelopmental disability. Whole transcriptome sequencing (RNA-Seq) offer insights into mechanisms underlying FASD, but gene-level analysis provides limited information regarding complex transcriptional processes such as alternative splicing and non-coding RNAs. Moreover, traditional analytical approaches that use multiple hypothesis testing with a false discovery rate adjustment prioritize genes based on an adjusted p-value, which is not always biologically relevant. We address these limitations with a novel approach and implemented an unsupervised machine learning model, which we applied to an exon-level analysis to reduce data complexity to the most likely functionally relevant exons, without loss of novel information. This was performed on an RNA-Seq paired-end dataset derived from alcohol-exposed neural fold-stage chick crania, wherein alcohol causes facial deficits recapitulating those of FASD. A principal component analysis along with k-means clustering was utilized to extract exons that deviated from baseline expression. This identified 6857 differentially expressed exons representing 1251 geneIDs; 391 of these genes were identified in a prior gene-level analysis of this dataset. It also identified exons encoding 23 microRNAs (miRNAs) having significantly differential expression profiles in response to alcohol. We developed an RDAVID pipeline to identify KEGG pathways represented by these exons, and separately identified predicted KEGG pathways targeted by these miRNAs. Several of these (ribosome biogenesis, oxidative phosphorylation) were identified in our prior gene-level analysis. Other pathways are crucial to facial morphogenesis and represent both novel (focal adhesion, FoxO signaling, insulin signaling) and known (Wnt signaling) alcohol targets. Importantly, there was substantial overlap between the exomes themselves and the predicted miRNA targets, suggesting these miRNAs contribute to the gene-level expression changes. Our novel application of unsupervised machine learning in conjunction with statistical analyses facilitated the discovery of signaling pathways and miRNAs that inform mechanisms underlying FASD.

## Author summary

Genomic research often yields an overwhelming amount of information. Accurate models for predicting and validating multivariate big data in genomics distill complex relationships and interactions. A prime example is fetal alcohol spectrum disorders, the largest known cause of neurodevelopmental disability affecting nearly 5% of children in the United States. Alcohol exposure during pregnancy leads to complex epigenetic and transcriptomic modifications, subsequently impairing signaling pathways in neural and morphologic development. Identifying transcriptomic mechanisms regulating alcohol’s teratogenicity during embryonic development is crucial for understanding variable phenotypic outcomes. This allows for the advancement of future therapeutic interventions that may mediate alcohol’s effects. Most genomic studies do not incorporate various levels of transcriptomic analysis, spanning gene, exon, and splicing variants, because it is difficult to meaningfully consolidate all those analyses. Therefore, enhancing machine learning approaches that corroborate traditional statistical methods can yield novel relationships, and is important for robust functional experiments that proceed from such genomic studies.

## Introduction

Transcriptome-level approaches such as RNA-Seq capture an expression-level snapshot of an experimental system. RNA-Seq is an important discovery platform that generates insights for targeted hypothesis development and testing. However, gene-level analysis provides limited insight into transcriptomic regulation, in part because analytical tools often exclude transcripts represented by splicing variants and altered exon representation [1]. Gene-level analyses can also misrepresent fold-changes. For example, a gene may have two upregulated and two downregulated exons, and thus yield in a net result of no fold-change difference in abundance between the treatment and control. Understanding these exon-level differences offers novel insights into regulatory mechanisms that are otherwise lost during gene-level analysis [1]. Additionally, statistical methods that emphasize transcript-level significance create a loss of information when prioritizing transcripts by their p-values.

When analyzing the big data sets that emerge from RNA-Seq, it is a cumbersome task to narrow down tens of thousands of exon targets to those having the greatest biological relevance. Although statistical models provide a system for condensing such information to the most statistically significant genes or exons, there often remains several thousand genes or exons having a false discovery rate (FDR) below 0.1 or even 0.05. Statistical cutoffs alone weakly inform how to prioritize or further narrow a still-extensive data set for follow-up analysis, and the biological importance of a gene or exon does not always correlate with the strength of the p-value. Thus, a more comprehensive approach is necessary to corroborate the statistical findings and thereby reduce, e.g., an RNA-Seq dataset of 80,000 exons to 5–10 candidate genes/exons for functional analysis. One approach is to employ the combination of statistical and machine learning methods to optimize a solution to the most likely biologically relevant information. By utilizing statistical inference approaches in conjunction with the mathematical models provided in unsupervised machine learning algorithms, the rigor of the two models introduces less bias when selecting the few genes/exons of interest. When applying an unsupervised machine learning model to a highly dimensional dataset, such as that from RNA-Seq, mathematically hidden patterns are learned by the algorithm that are otherwise not identifiable by the researcher [2]. Developing such methodologies is essential for analyzing and interpreting transcriptomic responses to an intervention or stressor.

One such stressor is alcohol. Prenatal alcohol exposure (PAE) is a leading source of neurodevelopmental disability that affects 3% to 5% of U.S. first-graders [3]. Its clinical manifestation, fetal alcohol spectrum disorder (FASD), is typified by growth reduction, deficits in learning and executive function, and a distinct facial appearance [4]; the latter is due to alcohol’s disruption of the neural crest progenitors that form the facial elements [5]. Alcohol alters cellular activity through its direct binding of hydrophilic pockets in select proteins that regulate intracellular signaling; the downstream transcriptional changes enable alcohol to redirect cellular function and fate [6]. Some of these transcription-level changes are mediated by traditional signaling effectors including β-catenin and sonic hedgehog [5]. Other transcriptional changes result from changes in DNA methylation and the altered abundance of both long non-coding RNAs and non-coding micro-RNAs, or miRNAs [7–9]; these latter may have diagnostic utility as biomarkers for alcohol-exposed infants. Using an embryonic avian model that recapitulates the facial deficits that occur in FASD, we utilized RNA-Seq to identify gene expression patterns that potentially inform how alcohol disrupts the development of these neural crest facial progenitors [10,11]. Our gene-level analyses of alcohol-exposed early neural folds, which are enriched in neural crest, identified 3422 transcripts having differential expression in response to alcohol, and these mapped to KEGG pathways with enriched representation including ribosomal biogenesis, oxidative phosphorylation, and mTOR, among others [10]. To gain additional insight into these transcriptional changes and their underlying mechanisms, here we apply principal component analysis (PCA) and k-means in conjunction with statistical approaches to optimize the discovery of functional exon transcripts. Unsupervised machine learning reduced the biological noise and dimensions of our multivariate RNA-Seq data to a subset of orthogonal variables that best defined the variance among the exon transcripts. This approach identified candidate splicing variants and regulatory motifs that shape cellular responses to alcohol.

## Methods

### Dataset

The RNA-seq dataset was originally described in Berres et al. [10]. To summarize, it was derived from neural fold-stage (4–7 somites) chicken (*Gallus gallus*, strain Special Black) embryos that were exposed to a pharmacologically-relevant alcohol concentration (52 mM for 90 min) or isotonic saline, followed by a 4.5 hr recovery period. The cranial headfolds were isolated 6 hours following the initial alcohol exposure. Following RNA isolation, cDNA synthesis, and quality assurance [10], paired-end reads (75 bp) were generated on an Illumina Genome Analyzer IIx (University of Wisconsin Biotechnology Center, Madison, WI). The obtained reads were freshly analyzed using the pipeline described below.

### Exon mapping and quantification

**Fig 1:**
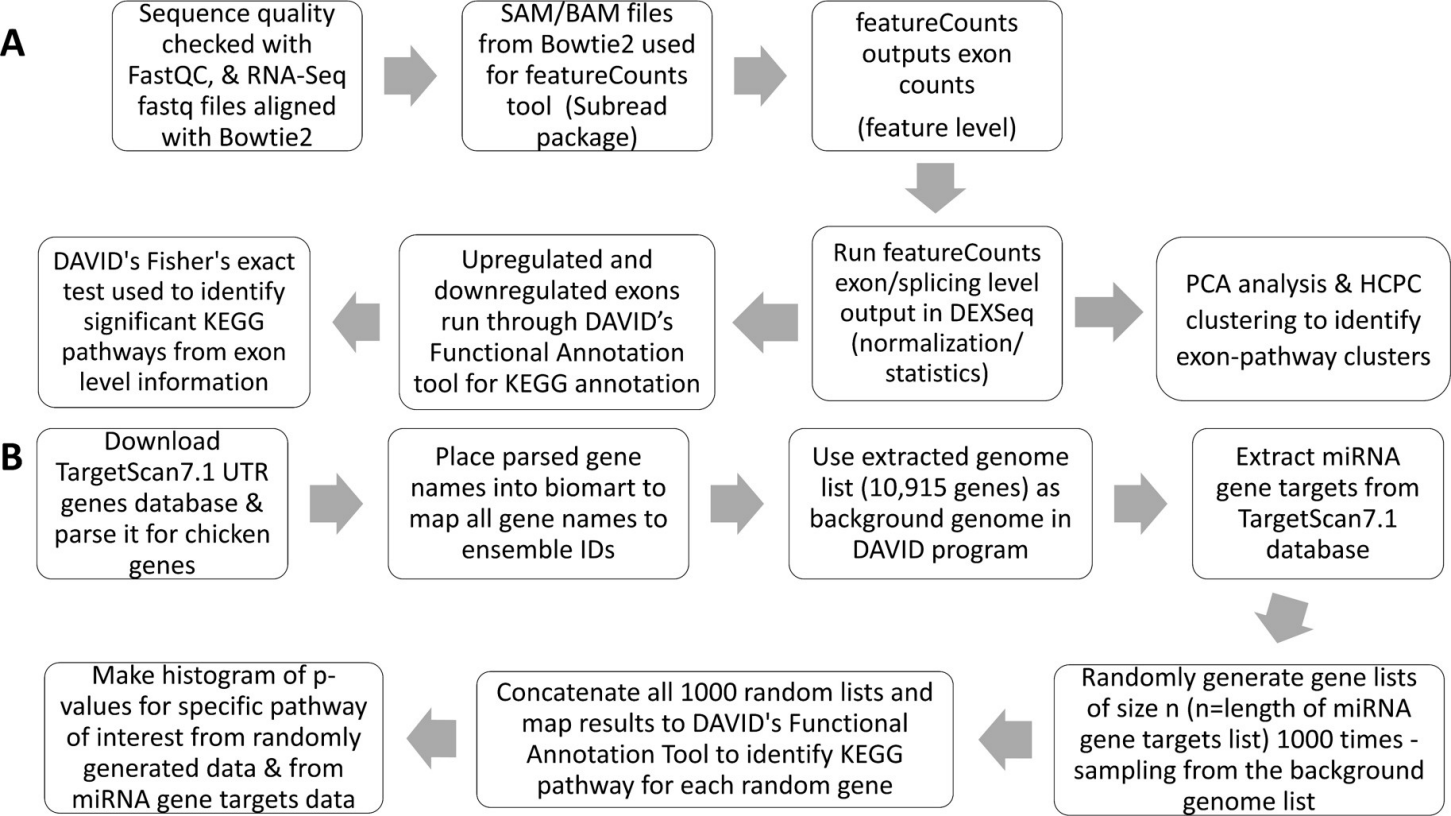
Exon Quantification and RDAVID Workflows. (A) The pipeline portrays the steps and tools used to map the raw sequence reads and quantify exon expression counts. (B) The creation process for the RDAVID program.

A schematic of the exon analysis pipeline is shown in Fig 1A. Trimming of the RNA-Seq sequence reads was performed as described in our previous study [10]. The quality of the fastq files was checked in FastQC to ensure there were no overabundant sequences, adapters, or poor sequence quality scores [12]. The fastq sequence files were then aligned to the *Gallus gallus* 5.0 Ensembl reference genome using Bowtie2 [13], and the resulting SAM file was converted to a BAM file and fed into the subread featureCounts package [14]. Parameters used in the subread featureCounts program included a stringency parameter (-B) that ensured the mapping of both paired ends when assigning a count to a specific exon. Additionally, the parameter -f was used to specify feature (exon) level counts and not metafeature (gene) level counts. Other unique parameters include -p and -s 0, which correspond to paired end runs and mapping reads either on the forward or reverse strand, respectively. For normalization and statistical analysis, we used the DEXSeq package under the R software v3.4.4. DEXSeq is a conservative approach that utilizes a negative binomial distribution with a generalized linear model to adjust for false positive significance values that result from running statistical tests on tens of thousands of exons [1]. The output of the featureCounts program was used as the input for the DEXSeq package. The DEXSeq analysis generated Benjamini-Hochberg (BH) adjusted p-values, log2 fold changes, exon base mean expression values, exon usage coefficients, normalized raw counts for each exon, exon coordinates, full and reduced linear regression model statistics, exon transcripts, and exon dispersion estimates, as detailed in the DEXSeq manual [1]. All subsequent statistical and machine learning analyses only utilized BH adjusted p-values below 0.1, or a false discovery rate (FDR) of 10%. Due to the DEXSeq analysis pipeline, it is important to note that negative fold changes correspond to genes upregulated by alcohol, and positive fold changes are genes downregulated by alcohol.

### Principal component analysis and HCPC clustering

We isolated upregulated exons and downregulated exons based on the log2 fold change and mapped each exon to its corresponding KEGG pathway(s) using the DAVID Functional Annotation Tool v6.8. We applied a principle component analysis with the exon IDs as the observations (~7,000 exons), and all the normalized raw counts and exon usage coefficients as the variables. All exons used in the PCA analysis had a BH adjusted p-value below 0.1 from the DEXSeq analysis. The KEGG pathways were used as the supplementary qualitative variable for identification of exon-pathway clusters. We scaled the data using the *FactoMineR* PCA package to standardize expression values that were measured in different scales (i.e. exon usage coefficients and read counts). A scree plot, a squared cosine quality of representation, and a correlation circle were generated using the *factoextra* R package. The contribution of an observation to the principal components was calculated using the *FactoMineR* package, where the squared factor score of the observation was divided by the eigenvalue of the component, resulting in the ratio used for the contribution of an exon [15]. To identify clusters of interest within the dataset, we utilized the Hierarchal Clustering on Principal Components (HCPC) algorithm adapted from [16]. The HCPC algorithm under the *FactoMineR* package combines hierarchal and k-means clustering to partition the observations into the most likely clusters representative of a large multivariate dataset. The HCPC algorithm first uses the Ward criterion for hierarchal clustering on the principal components; the Ward criterion is based on multidimensional variance, making it appropriate for post-PCA clustering [17]. The number of clusters was then determined with initial partitioning of the hierarchal tree, followed by k-means clustering to optimize the final result [17].

In parallel, we performed a separate cluster analysis of the exon dataset using a self-organizing map (SOM) / artificial neural network (ANN) approach. The optimal number of clusters to partition the dataset was calculated with the wss metric, gap statistic, and silhouette methods. We utilized the Kohonen R package and built our SOM with a 0.01 learning rate, 15x10 map, and 15,000 epochs. The number of epochs was calculated at the threshold in which the mean distance between an observation and the closest unit neuron decreased and remained stable. The dimensions of the SOM grid were calculated at the point in which the node counts map had a minimized number of empty nodes in the ANN. We then applied hierarchal clustering to view the partitioned clusters.

### miRNA PCA and k-means clustering

To identify the most likely functionally-relevant miRNAs, we conducted a PCA and k-means clustering analysis on those miRNA-encoding exons having BH adjusted p-values below 0.1. The exon-miRNA loci were identified using the UCSC *Gallus gallus* 5.0 genome browser and included miRNAs that were encoded within a single exon, across two exons, or were within an intron for which the flanking exons were significantly altered. We used miRbase (release 22) to confirm that all these miRNAs were previously validated in vivo [18–20]. The principal component analysis was conducted in the same manner as described above. For the k-means clustering analysis, we utilized the *factoextra* R package. The optimal number of clusters was determined using the elbow method, in which the within-sum of squares (wss) measure was plotted for each value of k clusters (1–10). The cutoff of k = 3 was based on the location of the bend in the plot (S1 Fig) where the wss measure leveled off and did not add substantial value to the variance explained by the clustering [17]. Calculating the optimal number of clusters using the average silhouette and gap statistic methods yielded an optimal number of k = 2 and k = 4 clusters, respectively, and taking the average between all three methods replicated the k = 3 clusters found with the wss metric. When generating the k-means graph, we ran the algorithm with 50 different random starting points, and the result with the lowest within-cluster variation was selected [17]. The ellipsis drawn around each miRNA cluster was calculated with Euclidean distance. To further validate the miRNAs found in the k-means clusters, we also applied a fuzzy c-means clustering to the dataset. Each miRNA was assigned a probability of belonging to each cluster (membership coefficient). To test for spatial randomness in the data, we assessed the cluster tendency of the miRNA dataset versus a randomly generated dataset using the Hopkins statistic, with a null hypothesis that the dataset was uniformly distributed, and the clustering was due to random chance [21]. A Hopkins statistic (H) of 0.5 means the data are uniformly distributed because the summations of the mean nearest neighbor distances in the real $\sum_{i=1}^{n}x_{i}$ and randomly generated $\sum_{i=1}^{n}y_{i}$ datasets are close to each other [21].

$$H=\frac{\sum_{i=1}^{n}y_{i}}{\sum_{i=1}^{n}x_{i}+\sum_{i=1}^{n}y_{i}}$$ (1)

Each randomly generated dataset contained the same number of n observations and similar numeric ranges as the exon variables in the miRNA dataset. We also performed a visual assessment of cluster tendency (VAT) using the *factoextra* R package by computing an ordered dissimilarity matrix with a Euclidean distance measure between miRNAs in the dataset. A VAT heatmap was also generated from the random dataset. The SOM neural network analysis could not be applied to the miRNA dataset because it contained too few predictors (N = 6) and observations (N = 22) for it to build a SOM map that contained enough observations per node. This prevented the SOM from properly learning the dataset as it could not reduce the mean distance errors between the observations and the ANN nodes.

### miRNA RDAVID pathway analysis

We identified each miRNA’s gene targets from the TargetScan 7.1 *Gallus gallus* database, and mapped each gene target name to its Ensembl ID using Ensembl’s biomart tool. We ran the resulting Ensembl ID gene list for each miRNA through DAVID’s Functional Annotation Tool v6.8 to identify the KEGG pathway clusters shared among the gene targets. In addition, we downloaded the TargetScan 7.1 UTR Sequences database and parsed the gene names belonging to the chicken species ID (9031). We placed the parsed gene names into biomart and mapped all gene names to their Ensembl gene IDs. This Ensembl gene IDs list (10,915 genes) was used as the background genome list in the RDAVID program. Using an approach similar to [20], we developed the RDAVID program, a custom-built R program (github.com/abrar-alshaer/RNA-Seq) that we used to evaluate the likelihood of the miRNA gene targets’ KEGG clusters resulting from random chance. We used the *RDAVIDWebService* library to programmatically access the DAVID API. In the program, we generated random gene lists of size n (n = same length of miRNA gene targets list) by sampling from the background genome list. This process was repeated 1000 times to create 1000 random gene lists of size n. Next, we concatenated all 1000 random lists and mapped the genes to DAVID's Functional Annotation Tool to identify the KEGG pathway clusters and their corresponding p-values. The p-values were obtained using Fisher’s exact test implemented by the DAVID software [22] and plotted by histogram. A schematic of the RDAVID pipeline is shown in Fig 1B.

## Results

### Dimension reduction identifies differentially expressed exons and pathways

Using the pipeline depicted in Fig 1A, we identified 6,857 exons that had significant differential expression in the comparison of control and alcohol-exposed cranial neural folds. Of these, 4,586 were increased and 2,271 were decreased in response to alcohol challenge (GEO accession: GSE115383). These 6,857 exons represented 1,251 genes, as compared with the 3422 genes we identified as alcohol-responsive when analyzed at the gene level [10]. Of these, 391 genes overlapped between the exon-derived and gene-derived lists (S1 Table). Our initial principal component analysis (PCA) attempted to identify pathway-gene interactions at the exon level. However, DAVID’s functional annotation tool does not specifically annotate exons with KEGG classifications; thus, when providing DAVID with an exon list that contains repeated gene IDs, it mapped the repeated genes (i.e. exons of one gene) as KEGG classifications with higher fold-enrichments. This led to a hyper-annotation of each exon in which multiple KEGG pathways mapped to incorrect fold-enrichments, further increasing the dataset’s dimensionality and variance such that exon-pathway clusters could not be identified. To resolve this, and because many of the KEGG pathways are subsets of larger biological processes, we instead assigned the DAVID-defined KEGG classifications to one of twenty meta-KEGG clusters based upon biological function, as described in S2 Table, and then repeated the PCA from the DEXSeq output. Analysis of the data using a scree plot, squared cosine quality of representation, and correlation circle affirmed the quality of the algorithm and confirmed that the PCA captured almost all the variance in the dataset within the first and second principal components.

**Fig 2:**
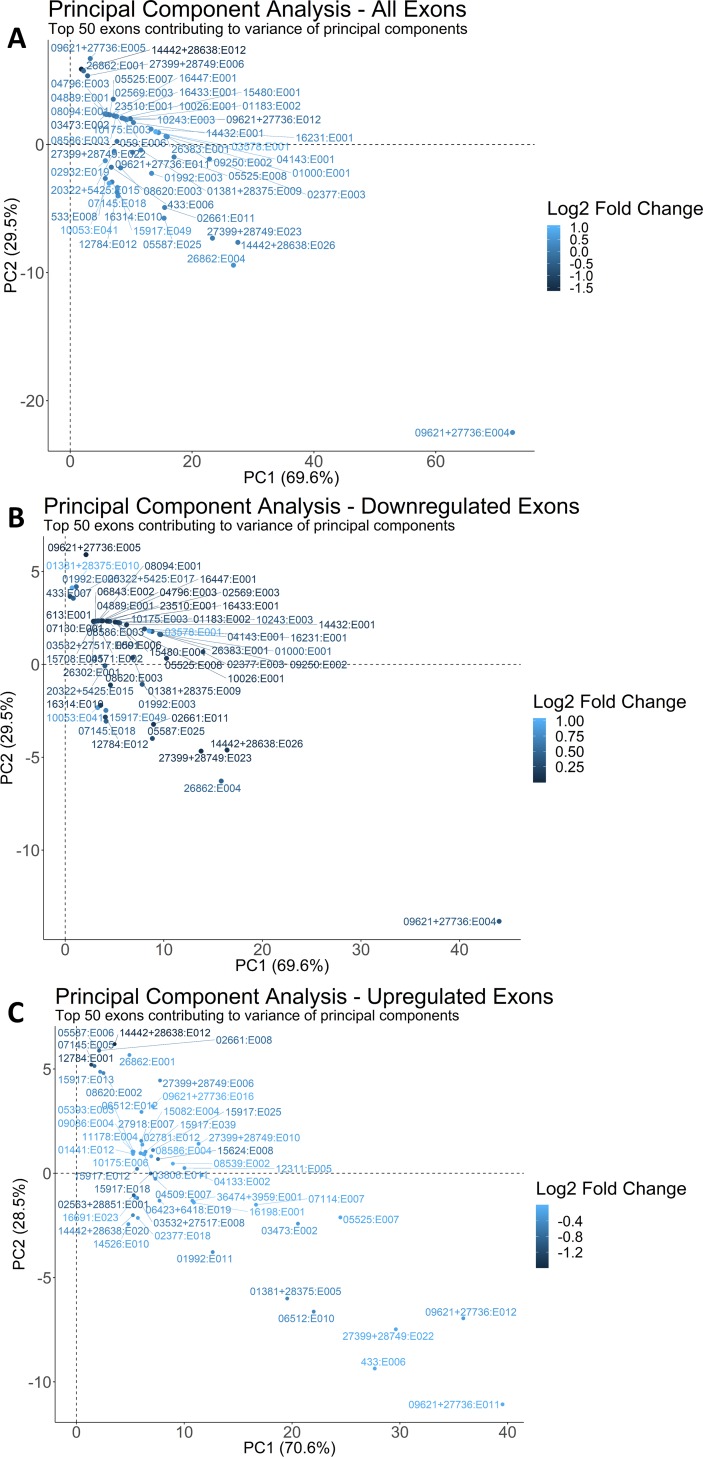
Exon-level principal component analyses. Exons farthest from the origin are the most differentially expressed transcripts. Note that positive fold-changes are down-regulated exons, and negative fold-changes are up-regulated exons. The common identifier (“ENSGALG0000”) for all the exon Ensembl IDs in the PCA plots was removed for legibility. (A) PCA of the top 50 exons contributing to the variance of the dataset, irrespective of fold-change direction. (B) PCA of the top 50 exons contributing to the variance of the dataset that were down-regulated by alcohol. (C) PCA of the top 50 exons contributing to the variance of the dataset that were up-regulated by alcohol.

The top 50 unique exons IDs contributing to the variance of the principal components (PC), regardless of fold-change, are plotted in Fig 2A and presented in Table 1, which also includes the contribution of each exon to the variance in dimensions one and two of the PCA. Of these top contributing 50 exons, 40 were downregulated in response to alcohol and only one exon (within SFRP1) was upregulated with a log2 fold change greater than one. Expansion of this analysis to the top 100 or even top 200 exons did not provide additional information to the PCA above that of the top 50, and again, only 2 of the 100 were upregulated with notable log2 fold changes (at least greater than 1). These 50 exons represented 42 genes, and of these 13 genes overlapped with the 3,422 genes previously identified in our gene-level analysis of this transcriptome set [10], two of which were the ribosomal proteins RPL39 and RPS20 (Table 2). Several genes contributed multiple exons to these top 50 and included β-actin (ACTB, 4 exons), cytoplasmic-2-like actin (ACTG1, 3 exons), glyceraldehyde-3-phosphate dehydrogenase (GAPDH, 2 exons), and claudin-1 (CLDN1, 2 exons). A separate PCA on the top 50 down-regulated exons (Fig 2B) and 50 up-regulated exons (Fig 2C) similarly revealed that the up-regulated exons had a weaker impact on the PCA, as reflected in their lower contribution values to the first two dimensions. Thus, the exon-level analysis identified additional exon targets that were not discovered at the gene-level. Moreover, although only 33.1% of the differentially-expressed exons were down-regulated, the PCA results suggest that down-regulation was the most significant transcriptional response to alcohol.

**Table 1:** Top 50 contributing exon annotations from PCA.

Ensembl IDs	Gene Name	ALC 1	ALC 2	CONT 1	CONT 2	Log2 Fold-change	Dim.1	Dim.2
ENSGALG00000009621+ENSGALG00000027736.E004	gga-mir-3533	60206	57042	107686	108095	0.383	34.77	7.86
ENSGALG00000014442+ENSGALG00000028638.E026	Predicted miRNA	21886	22914	40694	40610	0.126	4.99	0.91
ENSGALG00000026862.E004	CLDN1	29340	26847	31613	31224	0.476	4.74	1.38
ENSGALG00000027399+ENSGALG00000028749.E023	Predicted miRNA	19249	20566	33739	34107	0.087	3.60	0.83
ENSGALG00000002377.E003	ENO1	19212	17021	30925	30494	0.292	3.44	0.02
ENSGALG00000005525.E008	SRSF1	15783	14124	21303	20834	0.150	1.91	0.01
ENSGALG00000005587.E025	EIF4G2	18157	15096	18473	17863	0.282	1.56	0.51
ENSGALG00000002661.E011	YWHAE	14938	13492	21699	21302	0.117	1.58	0.38
ENSGALG00000009250.E002	HNRNPA3	14923	12503	18989	18961	0.265	1.67	0.01
ENSGALG00000026383.E001	TMSB4X	13396	12162	20079	20036	0.287	1.64	0.01
ENSGALG00000001000.E001	HSPA5	14983	13591	17537	17514	0.486	1.63	0.01
ENSGALG00000004143.E001	YWHAB	13909	12048	16449	16297	0.394	1.39	0.01
ENSGALG00000003578.E001	FN1	11976	10427	17875	17976	1.016	1.30	0.02
ENSGALG00000001992.E003	PKM	10124	11979	18758	19086	0.369	1.17	0.08
ENSGALG00000016231.E001	DDX3X	13452	11169	14252	13950	0.333	1.16	0.02
ENSGALG00000001381+ENSGALG00000028375.E009	gga-mir-6703-201	9703	9424	15503	15508	0.199	0.89	0.00
ENSGALG00000014432.E001	RPS20	7785	6851	14378	14257	0.151	0.71	0.05
ENSGALG00000009621+ENSGALG00000027736.E011	ACTB	9356	9268	12715	12592	-0.055	0.69	0.01
ENSGALG00000009621+ENSGALG00000027736.E012	ACTB	8584	8876	10578	10579	-0.220	0.64	0.06
ENSGALG00000010243.E003	PRDX1	7882	6544	11483	11615	0.384	0.57	0.06
ENSGALG00000001183.E002	MYH10	7826	7216	10228	9881	0.189	0.52	0.06
ENSGALG00000007145.E018	ITGB1	8665	7280	11728	11337	0.488	0.41	0.25
ENSGALG00000010026.E001	PPP1CB	8086	6783	9412	9199	0.149	0.48	0.07
ENSGALG00000015480.E001	POGLUT1	8178	7690	8372	8437	0.147	0.48	0.07
ENSGALG00000000059.E006	F1NMU4	6156	7567	9951	10048	0.086	0.47	0.07
ENSGALG00000012784.E012	TXNDC5	9117	7759	10336	10044	0.230	0.39	0.22
ENSGALG00000008620.E003	RPL39	7507	6445	12364	12464	0.192	0.45	0.05
ENSGALG00000015917.E049	EEF1A1	5881	5842	14080	14018	0.684	0.40	0.18
ENSGALG00000016433.E001	YWHAQ	6672	5826	8844	8719	0.068	0.38	0.07
ENSGALG00000005525.E007	SRSF1	6304	5409	6898	6941	-0.103	0.33	0.19
ENSGALG00000010175.E003	HSP90AB1	6000	5500	9377	9444	0.400	0.38	0.07
ENSGALG00000008586.E003	TUBB4B	5872	5545	9139	9418	0.303	0.37	0.07
ENSGALG00000027399+ENSGALG00000028749.E022	Predicted miRNA	6400	6739	9856	9926	-0.092	0.39	0.00
ENSGALG00000023510.E001	ERP29	6484	6158	7445	7575	0.093	0.34	0.08
ENSGALG00000009621+ENSGALG00000027736.E005	gga-mir-3533	1446	1349	2016	1972	0.018	0.07	0.70
ENSGALG00000016314.E010	ELOVL5	7934	6799	9228	9054	0.158	0.31	0.13
ENSGALG00000020322+ENSGALG00000005425.E015	OGT	6575	5831	10038	9909	0.386	0.34	0.00
ENSGALG00000010053.E041	PTPRF	6856	6633	9136	8881	0.868	0.27	0.14
ENSGALG00000000433.E006	TUBA4A	5630	7173	9327	9456	-0.065	0.30	0.05
ENSGALG00000016447.E001	PDIA6	5609	5007	7429	7250	0.206	0.28	0.08
ENSGALG00000002569.E003	RAN	4947	4427	8100	8332	0.141	0.27	0.08
ENSGALG00000004796.E003	CDC42	5763	5145	6269	6601	0.084	0.26	0.09
ENSGALG00000000533.E008	SRSF3	6019	5564	8675	8633	0.130	0.22	0.11
ENSGALG00000003473.E002	SFRP1	6248	5826	4652	4601	-0.249	0.23	0.09
ENSGALG00000004889.E001	SLC2A1	4731	4558	6590	6542	0.237	0.22	0.09
ENSGALG00000014442+ENSGALG00000028638.E012	Predicted miRNA	587	554	310	329	-1.567	0.02	0.54
ENSGALG00000008094.E001	HSPD1	4748	4159	6670	6485	0.261	0.21	0.09
ENSGALG00000026862.E001	CLDN1	849	1101	673	672	-0.222	0.03	0.51
ENSGALG00000027399+ENSGALG00000028749.E006	Predicted miRNA	1357	1608	1766	1911	-0.372	0.05	0.45
ENSGALG00000002932.E019	NME2	4809	4473	9393	9326	0.421	0.22	0.03

The Ensembl gene IDs, gene names, normalized exon counts for both pooled samples in alcohol-treated (ALC) and control (CONT) groups, log2 fold-changes, and contribution calculations of the first two dimensions for each exon in the PCA.

**Table 2:** Shared gene targets between exon-level (Top 50) and gene-level analysis.

Gene ID	Exon	Gene	Wikigene name	Gene-level Log2 Fold-change (Alc/Cont)	Gene-level P adjusted	Exon Level Log2 Fold-change (Cont/Alc)	Exon Level P adjusted
ENSGALG00000001381	E009	ACTB	ACTG1	0.78447	1.60E-02	0.19924	4.11E-17
ENSGALG00000002932	E019	NME2	NME2	0.60924	2.45E-08	0.42073	0.00933
ENSGALG00000003473	E002	SFRP1	SFRP1	1.46779	4.76E-05	-0.24910	2.99E-06
ENSGALG00000005587	E025	EIF4G2	EIF4G2	1.24812	2.68E-02	0.28156	0.00235
ENSGALG00000007145	E018	ITGB1	ACTG1	0.78447	1.60E-02	0.48766	1.94E-16
ENSGALG00000008620	E003	RPL39	RPL39	0.63949	5.18E-02	0.19245	5.63E-06
ENSGALG00000009250	E002	HNRNPA3	LOC100859627	0.74848	2.08E-02	0.26493	0.01029
ENSGALG00000009621	E004	gga-mir-3533	ACTB	0.73941	1.17E-03	0.38293	8.64E-19
	E005					0.01755	0.00119
	E011					-0.05466	4.42E-13
	E012					-0.22022	7.80E-11
ENSGALG00000010026	E001	PPP1CB	PPP1CB	1.35157	4.21E-03	0.14897	7.45E-09
ENSGALG00000014432	E001	RPS20	RPS20	0.61603	1.71E-03	0.15055	4.36E-10
ENSGALG00000014442	E012	GAPDH	GAPDH	0.65804	2.58E-06	-1.56687	1.56E-45
	E026					0.12600	1.44E-09
ENSGALG00000026862	E001	CLDN1	CLDN1	1.31928	2.32E-02	-0.22246	0.03191
	E004					0.47578	0.00590
ENSGALG00000028749	E006	ACTG1	LOC776816	0.75370	8.21E-03	-0.37219	0.00150
	E022					-0.09153	0.00682
	E023					0.08719	6.87E-05

Top 50 exon identities that overlap with gene-level targets from [7], including Ensembl gene ID, exon locus, Ensembl gene name, Wikigene name, gene-level log2 fold-change, gene-level Benjamini-Hochberg (BH) adjusted p-value, exon level log2 fold-change, and the exon level BH adjusted p-value.

When we instead used a self-organizing map (SOM) approach to cluster our exon-level data, the results replicated our PCA analyses findings and again partitioned the miRNA-encoding exon in ACTB farthest from all other exons (S2 Fig). SOM of the down-regulated and up-regulated exons also generated clusters that were similar to those from the PCA. When we then partitioned the SOM into various clusters using hierarchal clustering, we again replicated the HCPC results (hierarchal clustering on principal components). However, this approach did not add additional information above that obtained from the k-means and HCPC approaches.

To place the differentially-expressed exons into a cellular context, and to gain insight into how exonal choice might have emerged, we then asked which KEGG pathways were represented by these top 50 up-regulated and down-regulated exons. We again used the meta-cluster KEGG approach as above, due to the exon annotation limitations of DAVID. In both sets, the most frequently represented KEGG meta-cluster controlled cellular metabolism and encompassed KEGG pathways including mTOR signaling, autophagy, and nitrogen metabolism (32 exons up; 26 exons down; Table 3). Other enriched clusters included stress response (18 up, 22 down), cell adhesion molecules (14 up, 23 down), ribosomal biogenesis (15 up, 10 down), and TCA/oxidative phosphorylation (10 up, 8 down). The ribosome and oxidative phosphorylation KEGG clusters were identified in our previous gene-level analysis [10], whereas mTOR signaling (p = 0.41; 17 genes) and focal adhesion (p = 0.078) did not achieve significance in that analysis. Within a cluster, the gene lists for both the up-regulated and down-regulated top exons frequently overlapped, and some genes harbored exons that alternately increased and decreased in response to alcohol. For example, seven genes overlapped within the mTOR/autophagy/nitrogen metabolism cluster (ACTC2L, ACTG1, EEF1A1, EIF4G2, ITGB1, TUBB4A, TUBB4B), six genes overlapped for cell adhesion (ACTC2L, ACTG1, CLDN1, ITGB1, TUBB4A, TUBB4B), and four genes overlapped for ribosome biogenesis (EEF1A1, EIF4G2, RPL39, SRSF1). Other genes were uniquely represented in the up-regulated (ACTB, BRCA1, HSPA8) and down-regulated (HSPA5, RAN, RAC1) exon sets. Thus, alcohol had complex effects upon exon choice within a given gene. Importantly, the PCA approach implicated novel pathways potentially influenced by alcohol, and independently validated those identified in our previous gene-level analysis.

**Table 3:** KEGG pathways of top 50 Up-regulated and down-regulated contributing exons.

**Pathways Upregulated by ETOH**			
*Meta-KEGG Pathway Group*	*Number of Exons*	*Number of Genes*	*Gene Symbols*
mTOR-autophagy-N metabolism	32	23	ACTB, TUBA4A, SRSF1, HSPA8, ACTG1, APOA1, SRSF10, ACTC2L, U2AF1, UBE2D3, EEF1A1, TUBB4B, RHOA, RPS6, EIF4G2, BRCA1, gga-mir-3064, RPL39, ITGB1, ENOPH1, UBE2G1, UBB, PDPK1
Stress response	18	13	ACTB, TUBA4A, ACTG1, UBE2G1, ENO1, AP1M1, HSPA8, ITGB1, TUBB4B, HSP90AB1, UBE2D3, TXNDC5, ACTC2L
Ribosomal Biogenesis	15	10	SRSF1, HSPA8, SRSF10, U2AF1, DDX5, EEF1A1, RPS6, EIF4G2, RPL39, PDPK1
Cell Adhesion Molecules	14	10	ACTB, ACTC2L, TUBA4A, ACTG1, PDPK1, VCAN, TUBB4B, RHOA, ITGB1, CLDN1
TCA/OxPhos	10	9	PKM, APOA1, ALG12, PDPK1, UBB, GAPDH, ENO1, TPI1, PAFAH1B2
FoxO	8	7	PKM, PDPK1, GAPDH, ENO1, YWHAE, RHOA, TPI1
Endocytosis	6	5	RHOA, ARF1, HSPA8, EHD3, TPM4
NC development	4	3	SFRP1, TPI1, SLC25A6
MAPK Signaling	2	1	HSPA8
1-Carbon metabolism	1	1	PKM
**Pathways Downregulated by ETOH**			
*Meta-KEGG Pathway Group*	*Number of Exons*	*Number of Genes*	*Gene Symbols*
mTOR-autophagy-N metabolism	26	23	gga-mir-3533, gga-mir-3064, HSPA5, RAN, PPP1CC, SLC2A1, SRSF1, EIF4G2, RAC1, ITGB1, TUBB4B, RPL39, HNRNPA3, PPP1CB, PTPRF, RPS20, EEF1A1, SRPRB, TUBA4A, F1NMU4, ACTG1, OGT, ACTC2L
Stress response	22	20	gga-mir-3533, ACTC2L, ACTG1, TUBB4B, F1NMU4, HSPA5, ENO1, PPP1CC, CDC42, NCBP2, RAC1, ITGB1, HSPD1, TUBA4A, PPP1CB, HSP90AB1, TXNDC5, DDX3X, PDIA6, ERP29
Cell Adhesion Molecules	23	20	gga-mir-3533, ACTC2L, ACTG1, OGT, F1NMU4, TUBA4A, TMSB4X, CLDN1, FGFR3, POGLUT1, PTPRF, PPP1CB, TUBB4B, ITGB1, RAC1, SLC2A1, CDC42, PPP1CC, FN1, MYH10
FoxO	13	11	OGT, GAPDH, PKM, ENO1, YWHAE, YWHAB, PPP1CC, SLC2A1, PPP1CB, PTPRF, YWHAQ
Ribosomal Biogenesis	10	10	gga-mir-3064, HSPA5, RAN, SRSF1, EIF4G2, RPL39, HNRNPA3, RPS20, EEF1A1, SRPRB
TCA/OxPhos	8	7	GAPDH, PKM, ENO1, CDC42, RAC1, PRDX1, ELOVL5
Cell cycle	5	4	PKM, YWHAE, YWHAB, YWHAQ
MAPK Signaling	3	3	CDC42, RAC1, FGFR3
Endocytosis	3	3	RAB11B, CDC42, FGFR3
Cardiomyocyte Signaling	2	2	PPP1CC PPP1CB
1-Carbon metabolism	2	1	PKM
NC development	2	2	CDC42, RAC1

The KEGG pathway groups, number of exons/genes in each pathway, and gene symbols for the top 50 contributing exons contained in that pathway that were upregulated/downregulated by alcohol. The composition of pathways within each meta-KEGG is presented in S2 Table.

### Cluster analysis identifies candidate miRNAs in response to alcohol

**Fig 3:**
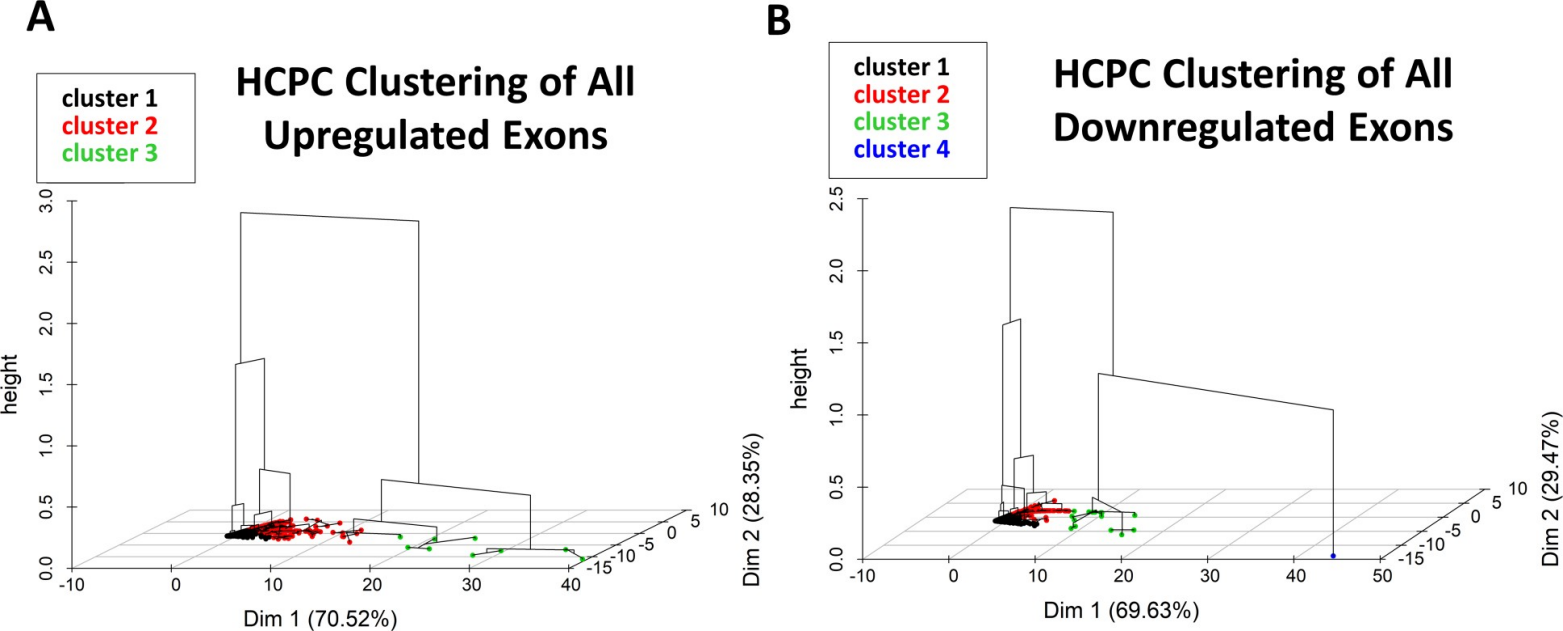
3D hierarchal representation of HCPC clustering. (A) Visualization of the HCPC clustering results for exons up-regulated by alcohol. (B) Visualization of the HCPC clustering results for exons down-regulated by alcohol.

**Fig 4:**
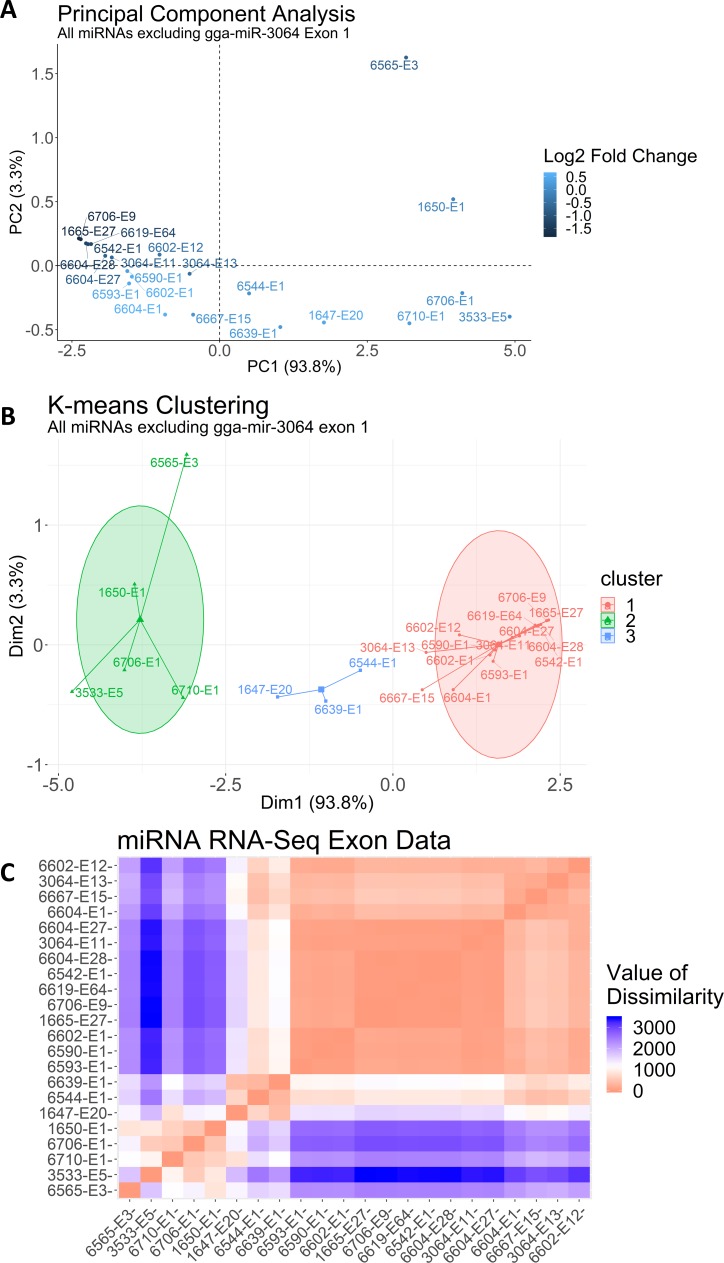
PCA, K-means, and VAT of miRNAs. The common identifier (“gga-miR”) for all the miRNA-exon Ensembl IDs was removed for legibility. All miRbase IDs in the plots are followed by a hyphen and the correspond exon number. (A) A PCA of the UCSC-verified miRNA-containing exons in our dataset (excluding gga-miR-3064 exon 1). Repeated miRNA IDs are due to a miRNA spanning more than one exon. Note that positive fold-changes are down-regulated exons, and negative fold-changes are up-regulated exons. (B) K-means clustering (k = 3 clusters) of all UCSC-verified miRNA exons, ellipses are drawn using Euclidean distance (excluding gga-miR-3064 exon 1). (C) A heatmap representation of the visual assessment of cluster tendency for all miRNA exons. Red corresponds to high similarity, and blue corresponds to low similarity.

Hierarchical clustering of principal components (HCPC) of all 6,857 exons for both the upregulated (N = 4,586; Fig 3A) and downregulated (N = 2,271; Fig 3B) sets identified several clusters and a single distinctive outlier within the downregulated exon set. That downregulated outlier was exon 4 of the β-Actin (ACTB) gene and is adjacent the microRNA gga-miR-3533 in exon 5. Further inspection of the PCA and HCPC results uncovered 30 unique exons with a BH p-value below 0.1 that encoded known or predicted miRNAs. Of these, 23 were mapped to the UCSC genome browser, and these represented 19 unique miRNAs. The abundance and annotation for these 23 exons is presented in Table 4. Most of these miRNA-encoding exons (70%) had a fold-change distinct from the gene-level change, suggesting these exons were differentially regulated. We used the 23 mapped exons to generate a PCA and k-means clustering analysis of the exon transcripts that contain miRNAs in the dataset, to identify which miRNAs explained the most variance among the PCs. Because some of the miRNAs were encoded across spliced exons, or were encoded within an intron, we included any exons that spanned the miRNA locus on the gene. In the first iteration, gga-miR-3064 exon 1 skewed all the other exons to one cluster due to its high normalized transcript abundance; thus, it was excluded from subsequent PCA and k-means clustering analysis. In the reanalysis (Fig 4A**)**, several miRNA-containing exons had high log2-fold changes and a farther distance from the origin, suggesting they may represent functionally relevant miRNAs because they explained more variance among the principal components.

**Table 4:** miRNA exons annotations.

Ensembl ID	miRBase ID	Gene Symbol	Gene Name	ALC 1	ALC 2	CONT 1	CONT 2	BH P-value*	Exon Log2 FC	Gene Log2 FC
ENSGALG00000025382+ENSGALG00000001043:E020	gga-mir-1647	REXO1	RNA exonuclease 1 homolog	611	713	893	949	0.004	0.449	-0.043
ENSGALG00000003532+ENSGALG00000027517:E001	gga-mir-3064	DDX5	DEAD-box helicase 5	3896	3546	6352	6185	1.08E-12	0.337	0.178
ENSGALG00000009621+ENSGALG00000027736:E005	gga-mir-3533	ACTB	Beta Actin	1446	1349	2016	1972	0.001	0.018	0.439
ENSGALG00000027916+ENSGALG00000008553:E001	gga-mir-6544	INO80	INO80 complex subunit	464	395	495	518	0.065	0.106	-0.194
ENSGALG00000042088+ENSGALG00000028980:E001	gga-mir-6590	ATG2B	autophagy related 2B	77	71	88	66	0.060	0.322	-0.681
ENSGALG00000030446+ENSGALG00000041520:E001	gga-mir-6593	CCDC94	coiled-coil domain containing 94	52	58	110	112	0.043	0.470	0.438
ENSGALG00000028549+ENSGALG00000000721:E001	gga-mir-6602	SLC41A1	solute carrier family 41 member 1	77	84	100	83	0.002	0.578	-0.655
ENSGALG00000026724+ENSGALG00000041331:E001	gga-mir-6604	RRP12	RRP12-like protein	93	116	252	270	0.0003	0.605	0.248
ENSGALG00000005516+ENSGALG00000028228:E001	gga-mir-6639	HEATR6	HEAT repeat containing 6	479	473	742	717	0.020	0.230	0.186
ENSGALG00000006144+ENSGALG00000026170:E015	gga-mir-6667	CDK10	cyclin dependent kinase 10	179	199	341	350	0.044	0.173	0.567
ENSGALG00000025785+ENSGALG00000008380:E001	gga-mir-6706	DGKZ	diacylglycerol kinase zeta	1190	1350	1598	1662	0.025	0.104	0.087
ENSGALG00000039830+ENSGALG00000034961:E001	gga-mir-6710	DHX30	DExH-box helicase 30	903	1088	1398	1385	4.65E-06	0.294	-0.0013
ENSGALG00000009476+ENSGALG00000025191:E001	gga-mir-1650	CDK6	cyclin dependent kinase 6	1464	1393	1336	1377	0.006	-0.141	-0.204
ENSGALG00000025345+ENSGALG00000028561:E027	gga-mir-1665	SZT2	SZT2, KICSTOR complex subunit	6	7	2	3	0.093	-1.776	0.282
ENSGALG00000003532+ENSGALG00000027517:E011	gga-mir-3064	DDX5	DEAD-box helicase 5	51	46	48	35	0.041	-0.632	0.178
ENSGALG00000003532+ENSGALG00000027517:E013	gga-mir-3064	DDX5	DEAD-box helicase 5	258	247	230	254	0.0002	-0.479	0.178
ENSGALG00000038017+ENSGALG00000032029:E001	gga-mir-6542	SRF	serum response factor	15	15	5	13	0.090	-1.221	0.241
ENSGALG00000025769+ENSGALG00000015451:E003	gga-mir-6565	ZNF462	zinc finger protein 462	1605	1486	776	757	1.90E-38	-0.729	-0.496
ENSGALG00000028549+ENSGALG00000000721:E012	gga-mir-6602	SLC41A1	solute carrier family 41 member 1	206	193	129	100	0.0007	-0.413	-0.655
ENSGALG00000026724+ENSGALG00000041331:E028	gga-mir-6604	RRP12	RRP12-like protein	13	11	6	10	0.072	-1.271	0.248
ENSGALG00000026724+ENSGALG00000041331:E027	gga-mir-6604	RRP12	RRP12-like protein	27	41	28	39	0.073	-0.742	0.248
ENSGALG00000030904+ENSGALG00000038881:E064	gga-mir-6619	LRP1	LDL receptor-related protein 1 precursor	26	19	11	9	0.074	-1.200	-0.024
ENSGALG00000025785+ENSGALG00000008380:E009	gga-mir-6706	DGKZ	diacylglycerol kinase zeta	8	9	5	1	0.081	-1.686	0.087

The Ensembl gene ID, miRBase ID, gene symbol containing the miRNA, gene name, normalized exon counts for both pooled samples in alcohol-treated (ALC) and control (CONT) groups, log2 fold-changes (FC), and BH adjusted p-value for miRNA-containing exons that mapped to the UCSC genome browser. Repeated miRNA IDs are due to a miRNA spanning more than one exon. Only exons with BH p-values below 0.1 were analyzed.

Under k-means clustering, the 23 miRNA-containing exons formed three distinct cluster groups (Fig 4B) that were primarily defined by shared transcript abundance. The five most abundant miRNA exons grouped into cluster 2, the three miRNA exons in cluster 3 were the next most abundant, and the fourteen miRNA exons in cluster 1 were the least abundant. We also applied a fuzzy c-means clustering and replicated the same results, except gga-miR-6667 (exon 15) belonged to cluster 3 instead of cluster 1, and gga-miR-3064 (exon 13) shared a membership coefficient near 50% between clusters 1 and 3. To test that the miRNA k-means clusters were not due to random chance, we performed a visual assessment of cluster tendency (VAT) for a random dataset and for our miRNA exons dataset (Fig 4C). As expected, miRNAs within a PCA-defined cluster also clustered together in the VAT. The VAT revealed additional cluster subgroups, such that gga.mir.3064 (exon 11), gga.mir.6667, and gga.mir.6604 were more interrelated, as were gga.mir.1665, gga.mir.6706 (exon 1), gga.mir.6619, gga.mir.6542, and gga.mir.6604 (exon 28). Certain miRNAs also maintained consistent relationships with miRNAs in another cluster; for example, in the VAT heatmap gga.mir.3533 shared an opposite relationship with all 14 miRNAs in cluster 1. This is consistent with the finding of gga-miR-3533 exon 4 as a distinct outlier from all other exons. These groupings suggest relationships within and between miRNAs that may inform their means of regulation or their functional relevance in response to alcohol exposure. We also evaluated the dataset’s spatial randomness using the Hopkins statistic (H). For a randomly generated dataset, H = 0.499 and was consistent with a uniformly distributed dataset that was clustered due to random chance. For the miRNA exons dataset, H = 0.15 and we rejected the null hypothesis and concluded that the clusters were not due to random chance.

### RDAVID miRNA pathway analysis

**Fig 5:**
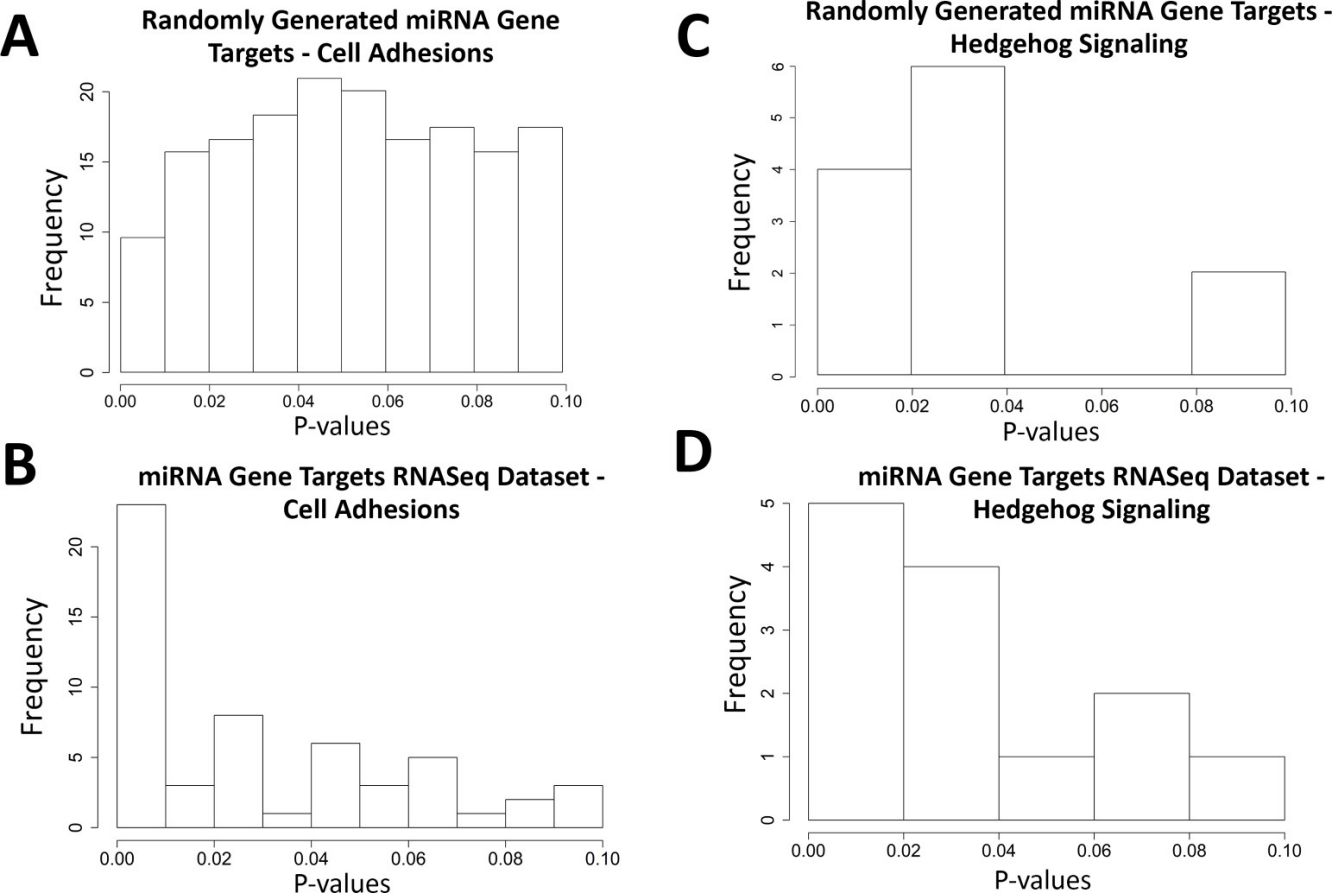
P-value distributions of RDAVID results. (A) A histogram of all p-values from the 1000 randomly generated miRNA gene targets in the cell adhesions pathway. (B) A histogram of all p-values from our RNA-Seq dataset’s miRNA gene targets in the cell adhesion pathways. (C) A histogram of all p-values from the 1000 randomly generated miRNA gene targets in the hedgehog pathway. (D) A histogram of all p-values from our RNA-Seq dataset’s miRNA gene targets in the hedgehog pathway.

To gain insights into the potential biological significance of these differentially represented exon-containing miRNAs, we used TargetScan7.1 to extract candidate gene targets for each individual miRNA and mapped these candidate genes into DAVID to identify the KEGG pathways most likely to interact with each miRNA. However, the TargetScan results are predicted targets and most have not been experimentally validated. To eliminate those KEGG pathways that arose from random chance due to false positive gene targets, and thereby identify pathways that were truly enriched, we first ran our RDAVID pipeline (Fig 1B) using a randomly generated gene target list of the same size (n = 1000) as the miRNA candidate gene targets list [20]. We repeated this 1000 times and mapped all random gene lists to their KEGG pathways; this did not require the meta-cluster approach because each miRNA represented a single gene. Histograms of p-values for two statistically significant KEGG pathways among the miRNA exons, cell adhesions (includes focal adhesion and cell adhesion molecules KEGG pathways) and hedgehog signaling, are presented in Fig 5. For cell adhesion, p-values for the randomly generated gene-pathways were uniformly distributed (Fig 5A), whereas those for the miRNA dataset were mostly below 0.01 (Fig 5B), suggesting the cell adhesion pathway enrichment was not due to false enrichment from the predicted targets. Parallel analysis of the next-most abundant pathways, insulin signaling and endocytosis, yielded similar results and suggested their emergence also did not represent false enrichment. In contrast, for the hedgehog signaling pathway, p-values for the randomly generated gene targets were not uniformly distributed and were similar to the distribution of p-values in our dataset (Fig 5C). This indicated that their presence in the miRNA data set (Fig 5D) likely represented a false discovery. The low abundance of these targets likely contributed to this type-1 error.

**Fig 6:**
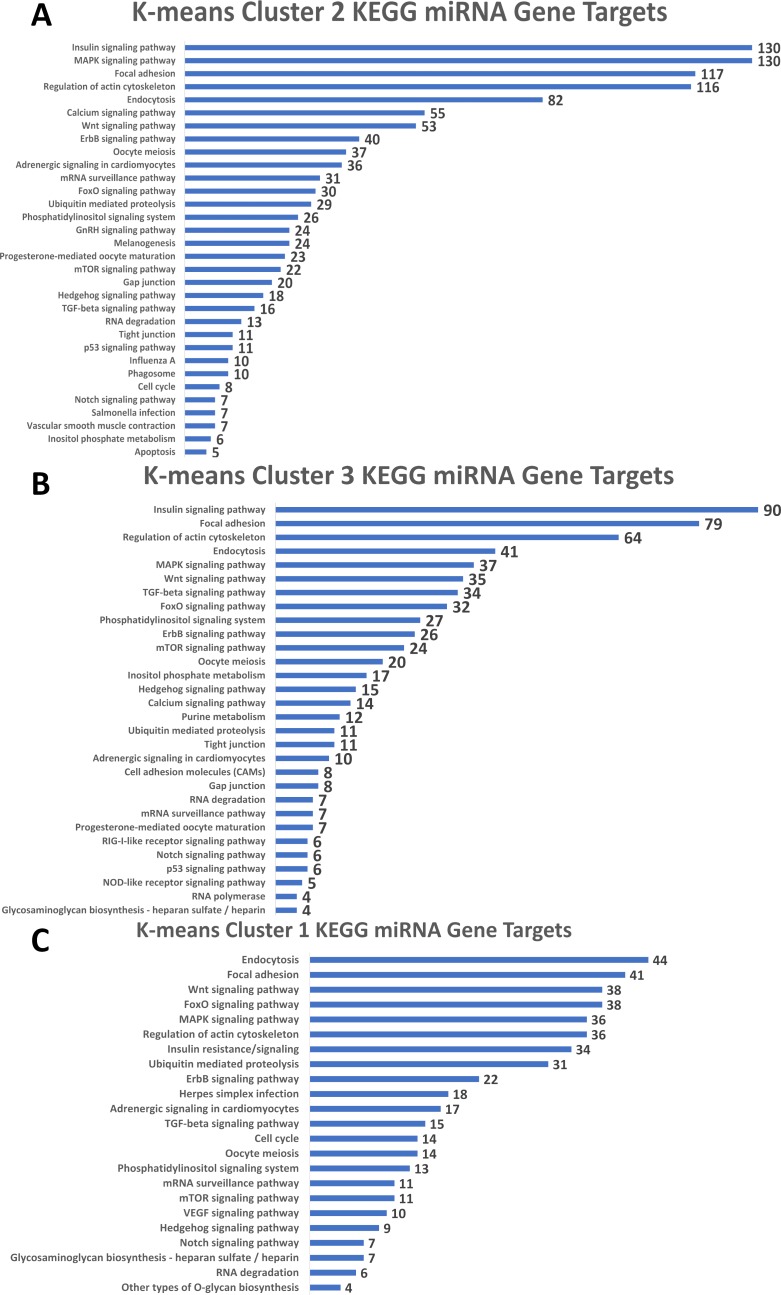
KEGG Representation for miRNA Clusters Identified through K-Means Analysis. (A) Listing of significantly enriched KEGG pathways in miRNA cluster 2, with the number of genes in each pathway indicated. (B) Listing of significantly enriched KEGG pathways in miRNA cluster 3, with the number of genes in each pathway indicated. (C) Listing of significantly enriched KEGG pathways in miRNA cluster 1, with the number of genes in each pathway indicated.

Following this RDAVID analysis, the most significantly enriched KEGG pathways that emerged from the candidate gene target list for each miRNA are presented in S3 Table. Several pathways emerged repeatedly, and the most commonly represented pathways included focal adhesion (15 miRNAs), regulation of actin cytoskeleton (12), insulin signaling (10), insulin resistance (9), MAPK signaling (9), and ErbB signaling (8). Although these pathways also were present within all three miRNA clusters identified using k-means, the three clusters differed in the specifics of their pathway enrichment. For the high-abundance miRNAs in cluster 2 (Fig 6A), the most enriched pathway targeted insulin signaling (128 genes) and several pathways crucial to neural crest development including MAPK signaling (128 genes), focal adhesion (117 genes), actin cytoskeleton (116 genes) and melanogenesis (23 genes). Other predicted pathways included known alcohol targets in this model including calcium signaling (55 genes) and Wnt signaling (53 genes) [5, 10]. The pathways in miRNA cluster 3 (Fig 6B) paralleled those of cluster 2 and similarly emphasized insulin signaling (90 genes), cell migration (focal adhesion, 88 genes; actin cytoskeleton, 64 genes), and MAPK signaling (37 genes), as well as Wnt signaling (35 genes) and TGF-β signaling (34 genes). MiRNAs in cluster 1 (Fig 6C) had the lowest abundance and a different target profile that emphasized endocytosis (44 genes), focal adhesion (41 genes), Wnt signaling (38 genes), MAPK signaling (36 genes), and regulation of actin cytoskeleton (36 genes). Overall, the differentially enriched miRNAs consistently targeted a limited set of pathways crucial for neural crest development. They also targeted pathways known to be dysregulated by alcohol in this cell population, implicating these miRNAs as candidate contributors to those mechanisms.

## Discussion

### Machine learning as a discovery tool for exome analysis

The most important finding from our study is that the use of exon level analysis in conjunction with unsupervised machine learning generates novel insights that were otherwise lost in gene-level analyses with statistics alone. Reliance on rankings of p-values can lead to prioritizing exons by significance, and this may not directly correlate with functional relevance. Such approaches in analysis of RNA-Seq data make it difficult to select exons of interest with the most likely biological relevance. While statistical tests are often effective at drawing inferences from a dataset, these inferences are based on assumptions from a given model that are likely to best fit the dataset in question [2]. Unlike statistical methods based on a conical model of inference, unsupervised machine learning assumes little to no information about the dataset in question. For example, principal component analysis (PCA) reduces the biological noise in the data set and identifies hidden patterns within and between exons to identify correlated variables. By implementing a principal component analysis (PCA), we reduced the dimensionality of our multivariate RNA-Seq dataset into 2–3 principal components that correspond to most of the variance in the data. To further identify relationships among the exons an unsupervised clustering algorithm, such as k-means, provides insight as to which exons share the closest mathematical distance, and this in turn can elucidate biologically relevant relationships.

An unexpected problem arose during the pre-processing of the exon data for implementation in the initial PCA analysis, and we discovered a limitation in the application of the DAVID functional annotation tool to exome analysis. Specifically, DAVID’s KEGG pathway annotations do not consider exon level mapping; hence, when assigning exons to KEGG pathways the annotation is collapsed to the gene ID and the KEGG becomes over-enriched. Moreover, since KEGGs are assigned by gene level information without specificity for exon transcripts or splicing variants, each exon receives all the KEGGs for all the exons associated with that gene. For example, if gene X had 10 exons, and gene X mapped to 45 KEGGs, each exon will receive all 45 KEGG annotations, resulting in 450 annotations for one gene (gene X). This creates hyper-annotation of KEGGs to each exon and significantly increases the error due to variance introduced into the machine learning algorithm. It is imperative to note that all machine learning approaches rely on the fine balance between the bias-variance tradeoff. Error due to variance results in underfitting of the machine learning algorithm where the model cannot adequately learn the dataset, therefore causing a higher probability of false prediction of exon classifications in the model [17]. Conversely, error due to bias results in overfitting of the dataset and may cause normally random trends in the dataset to be used as classification criteria in the model algorithm [17]. For this reason, we implemented the meta-KEGG approach to balance the bias-variance tradeoff in our PCA and k-means algorithms. This approach funneled the long list of KEGG annotations for each exon into 1–5 functional classifications, and this better balanced the ratio of bias and variance introduced into the algorithm with our dataset. We further enhanced the rigor of the machine learning models by utilizing only those exons with an FDR adjusted p-value below 0.1. Corroborating these statistical approaches with unsupervised machine learning enhanced the identification of novel differentially expressed exons.

In support of the validity of our machine learning approach, as this paper was finalized two new publications describe additional machine learning approaches to analyze RNA-Seq data with respect to alternately-spliced transcripts [23] and the prediction of genetic expression profiles [24]. However, the approach described in [24] utilizes supervised machine learning algorithms to predict environmental exposure from genetic and epigenetic expression profiles. Supervised methods were similarly used in [23] to predict healthy/disease phenotypes, tissue types, and other sample features. These methods differed from our approach as we utilized unsupervised algorithms for discovery of exome-level differential expression patterns, rather than feature prediction; therefore, without previous training of our model we limit assumptions about the RNA-Seq dataset. Application of an unsupervised neural network to the exon dataset obtained similar results; however, this approach was limited to the exome dataset due to the high number of observations.

### Machine learning at exome-level identifies alcohol-responsive candidate pathways

To our knowledge, this is one of a few handful of studies that directly compare gene-level and exon-level outcomes from RNA-Seq, and the first to study this with respect to alcohol exposure. Importantly, the exon-level analysis recapitulated major findings from the gene-level analysis and thus endorses the validity of this machine learning approach for exome discovery. Both approaches identified KEGG pathways representing ribosome biogenesis, oxidative phosphorylation, and spliceosome as having significantly altered enrichment in response to alcohol; expression in these pathways was suppressed [10,11]. However, the exon-level analysis generated a richer profile of these expression-level changes, and it captured additional pathways that only trended toward statistical significance in the gene-level analysis, including mRNA surveillance, cell cycle, and protein processing in the endoplasmic reticulum. The comparison of KEGG pathways for up-regulated exons and genes was less concordant and this was explained by the PCA, which found a weaker contribution of the upregulated exons to the data variance. Overall, the exon-level approach confirmed major findings from the gene-level analysis, and it highlighted that gene-level based interpretations do not fully capture the complexity of transcriptomic regulation. Several of these pathways (cell adhesion, regulation of actin cytoskeleton, cell migration, ribosome biogenesis, mRNA splicing) were also enriched in two independent microarray analyses of alcohol-exposed mouse neural folds having a parallel developmental stage [25, 26], endorsing that these changes represent conserved responses to alcohol across amniotes.

An unexpected finding from the exon-level PCA was the identification of a single exon, exon 4 within ACTB, which was highly differentiated from the other top 100 exons. This exon turned out to encode a miRNA, gga-miR-3533, and additional investigation revealed that many of the top contributing exons that responded to alcohol also encoded miRNAs. Our prior, gene-level analysis of this same data set identified miR-3533 but not these other sixteen miRNAs [10], likely because the statistical weighting approach minimizes significance when only a single exon or flanking exons is differentially expressed within that gene. Because RNA-Seq directly quantifies exome abundance, our approach enabled the retrospective capture of miRNAs from the same cDNA pool used to quantify mRNA, without the need for specialized extractions or microarrays selective for these short sequences. Endorsing this approach, the majority of these miRNA-encoding exons were differentially expressed relative to their parent genes, and thus were missed when the individual exon responses were collapsed during our prior gene-level analysis. MiRNAs are crucial regulators of gene expression. These small, non-coding RNAs average 21–23 nucleotides in size and typically target specific sequences within the 3’ end of mRNAs to effect translational repression or RNA destabilization; some miRNAs instead bind 5’ mRNA sequences to enhance translation and stability [27]. Because a single miRNA can target multiple transcripts, miRNAs are a powerful mechanism to rapidly redirect cellular activity at the translational level and at a low energetic cost. Non-coding RNAs including miRNAs are significant mediators of alcohol’s action, and they contribute to its pathological sequelae including neurotoxicity, teratogenicity, hepatotoxicity, inflammation, and to mechanisms of addiction, tolerance, and withdrawal [7, 28, 29]. MiRNAs also have clinical relevance to FASD, and a panel of miRNAs isolated from the maternal serum exosome strongly predicts the severity of birth outcomes in alcohol-exposed pregnancies [29]. The identification of alcohol-responsive miRNAs within the early cranial neural fold, which is vulnerable to alcohol’s neurotoxicity, is consistent with that work and extends the relevance of miRNA dysregulation to this early embryonic period.

The seventeen miRNAs having significantly altered representation in this model were independently validated in chick embryos including these stages [18, 20], but have not been previously identified as alcohol-responsive. While most of these miRNAs are unique to *G*. *gallus*, miR-3533 is also described for *B*. *taurus*, and miR-3064 is orthologous in multiple vertebrate species including human. Six of these miRNAs are located within genes linked with craniofacial and/or neurodevelopmental impairment when mutated in humans: ACTB, CDK10, CDK6, INO80, SZT2, and ZNF462 [30–35]. Three additional genes, DDX5, DHX30 and RRP12, participate in ribosome biogenesis [36–38]. Loss-of-function in ribosome biogenesis causes facial deficits [39] and is mechanistically linked to the alcohol-induced facial deficits and neural crest losses studied here [10]. DHX30 (MIM: 616423), which harbors miR-6710 in Exon 1, is an RNA helicase enriched in neural progenitors and is essential for mitochondrial ribosome biogenesis. DHX30 loss-of-function in mouse is embryolethal by day 9.5, and missense mutations within its core helicase produce intellectual disability and facial anomalies [37, 40]. A second RNA helicase, DDX5, also houses an alcohol-responsive miRNA, miR-3064, that is conserved across vertebrates. The DDX5 gene product, also known as p68 RNA helicase, mediates splicing of rRNA, mRNA, and miRNA, and it is a crucial transcriptional regulator [41]; it was also suppressed by alcohol in mouse neural folds [25]. Interestingly, miR-3064 has inhibitory interactions with mRNA encoding human telomerase reverse transcriptase, or hTERT [42], which promotes cell invasion by upregulating Snai2. Alcohol induces Snai2 in these cells to accelerate their epithelial-mesenchymal transformation [43], and the reduced miR-3064 observed here suggests a mechanism that might explain this response. Although these miRNAs have not been specifically linked to facial morphogenesis, their presence suggests an alternate means by which their domicile genes could influence cranial development.

The KEGG enrichments identified for these miRNAs largely replicates an independent functional mapping for these chick miRNAs [20], and many of these pathways are crucial for normal craniofacial development [44, 45]. Several of the pathways potentially targeted by these alcohol-responsive miRNAs mediate neural crest processes that are vulnerable to alcohol including cell cycle inhibition, apoptotic deletion, reduced induction, and altered migratory capacity [5, 46]. Furthermore, these enriched pathways were targeted by multiple miRNAs, and this redundancy suggests they are especially important for cellular alcohol responses. As one example, two frequently represented KEGG pathways were Focal Adhesion (#4510, 12 miRNAs) and Actin Regulation (#4810, 12 miRNAs). Cranial neural crest progenitors migrate from their dorsal neuroepithelial origin to occupy the ventrally positioned facial anlage, and alcohol reduces their migration by reducing focal adhesion formation and disrupting the actin cytoskeleton [5, 46, 47]. MiRNA-mediated regulation of cytoskeletal assembly offers a mechanism to explain how these migratory changes can persist long after the alcohol is cleared. Further contributing to these facial deficits is the widespread elimination of neural crest progenitors through alcohol’s activation of calcium-mediated apoptosis [5, 48, 49]. We showed previously that the pharmacologically-relevant alcohol level used here stimulates the rapid, G-protein-mediated release of intracellular calcium stores and activation of CaMKII within these cells, and blockade of this calcium transient or CaMKII fully prevents their alcohol-induced apoptosis [48, 49]; thus, enrichment for multiple miRNAs that may influence calcium signaling are consistent with this mechanism. The majority of these (gga-miR-6602-5p, gga-miR-6604-5p, gga-miR-6667-5p, gga-miR-6619-5p) target 5’ sequences in their predicted targets, suggesting an activating translational role [27] and a means by which these calcium signals could have a lasting impact upon these alcohol-exposed cells, and in complementation with CaMKII activation [49]. This calcium transient acts as a non-canonical Wnt signal through the CaMKII-mediated destabilization of nuclear β-catenin, and restoration of the latter’s transcriptionally activity rescues neural crest progenitors from alcohol-mediated apoptosis [5, 50]. Seven of the alcohol-responsive miRNAs target Wnt signaling (#4310), reflecting the Wnt pathway’s central role in neural crest survival, migration, and differentiation [44, 45]. Using a mouse neural crest model, Chen and colleagues showed that alcohol also activates p38 MAPK and decreases expression of miR-125b to stabilize p53 [51, 52]. While gga-miR-125b did not emerge from this analysis, nine of our miRNAs have enriched selectivity for MAPK signaling (#4010) and offers an additional means to modulate this pathway’s activity.

These miRNAs also offer novel insights into how alcohol alters neural crest development and survival. Signaling through ErbB governs neural crest pathfinding and migration, effected in part through downstream phosphorylation of Akt [53, 54], and loss of ErbB or its ligand neuroregulin disrupts neural crest migration and cranial ganglia and melanocyte development. Multiple alcohol-responsive miRNAs had predicted sequence specificity for pathways relevant to ErbB activity including ErbB signaling itself (#4012, 8 miRNAs), ten targeting insulin signaling (#4910, 10 miRNAs), and melanogenesis (#4916, 1 miRNA). Although ErbB is not a known alcohol target in the embryo, alcohol-ErbB interactions contribute to mammary oncogenesis [55] and its emergence here is consistent with alcohol’s well-documented suppression of neural crest migration [5, 47]. Also related to this is the identification of seven miRNAs linked to FoxO (#4068), a family of transcriptional effectors that operate downstream of ErbB/MAPK, as well as metabolic pathways including insulin and oxidative phosphorylation, to mediate cellular responses to stress. Alcohol upregulates FoxO in models of bone fracture healing [56] and intestinal barrier dysfunction [57], and its emergence here is consistent with that work. It may also further inform our β-catenin results, as FoxO can bind β-catenin to redirect and limit its Wnt transcriptional activity [58]. Taken together, alcohol’s dysregulation of multiple miRNAs in these neural folds is consistent with its pleiotropic actions and reflects its ability to interact with multiple proteins to redirect cellular activities. Despite this, the pathways enriched as potential miRNA targets represented a core set of signals crucial for normal development of these cells and known to be alcohol-responsive, either in this model or in other cell lineages. The internal consistency of these findings further validates machine-learning as an unbiased approach to elucidate alcohol mechanisms.

This work has several limitations, the most notable being that the aforementioned limitations in the DAVID software preclude exon-level KEGG pathway analysis, due to the multiplicative expansion of annotations when multiple exons per gene are represented. Although our work-around clustered the KEGGs into functional clusters and recapitulated our gene-level findings, it was also informed by that prior analysis. Further efforts in omics database management and annotation are needed to address this challenge. The other major limitation is that the predicted gene targets of these miRNAs identified in our *in silico* approach require functional validation. However, their relevance in this model is endorsed because these predicted pathways target known processes that are crucial to craniofacial morphogenesis and are known targets of alcohol. In conclusion, the application of statistical and machine learning algorithms to a complex exome dataset identified novel mechanistic candidates that were overlooked by approaches that emphasize p-value rank. It represents a method to distill the biological noise in a complex omic system and identify patterns that are otherwise missed, and its serves as a powerful tool for examination of exon/gene-pathway interactions.

## Supporting information

S1 Fig Optimal K-means clusters. The optimal number of clusters at k = 3 using the wss elbow method.
(TIF)

S2 Fig Artificial neural network self organizing maps on exon data. (A) A SOM was applied to the all exons with two partitioned clusters generated by the overlaid hierarchal clustering. (B) The training progress of the SOM model over 15,000 epochs for all exons. (C) A SOM was applied to the down-regulated exons with two partitioned clusters generated by the overlaid hierarchal clustering. (D) The training progress of the SOM model over 15,000 epochs for down-regulated exons. (E) A SOM was applied to up-regulated exons with three partitioned clusters generated by the overlaid hierarchal clustering. (F) The training progress of the SOM model over 15,000 epochs for up-regulated exons.
(TIF)

S1 Table GeneIDs for exon-level and gene-level analysis, and their intersection. Exon-level geneID list from this manuscript. List of geneIDs from the gene-level analysis is taken from Berres et al. (2017).
(XLSX)

S2 Table KEGG Groupings. The following table contains the clustered ‘meta-KEGG’ groups that were used to condense various KEGG pathways into clusters for the primary exon analysis. Note that a KEGG pathway can appear in multiple clusters, as dictated by biological function.
(DOCX)

S3 Table Significantly enriched KEGG clusters for each miRNA, as predicted using TargetScan. The unique miRNAs in our dataset and miRNA gene targets identified by the TargetScan7.1 database were placed into DAVID for KEGG enrichment analysis, from which the p-value for each pathway was obtained. Only p-values below 0.05 are reported, except for the focal adhesion pathway under gga-miR-3533-3p and all KEGG pathways of gga-miR-1665.
(DOCX)
