# Supplementary figures and images for: Exon level machine learning analyses elucidate novel candidate miRNA targets in an avian model of fetal alcohol spectrum disorder
Source: PLoS Comput Biol. 2019 Apr 11;15(4):e1006937. doi: 10.1371/journal.pcbi.1006937 (PMC6478348; doi:10.1371/journal.pcbi.1006937)

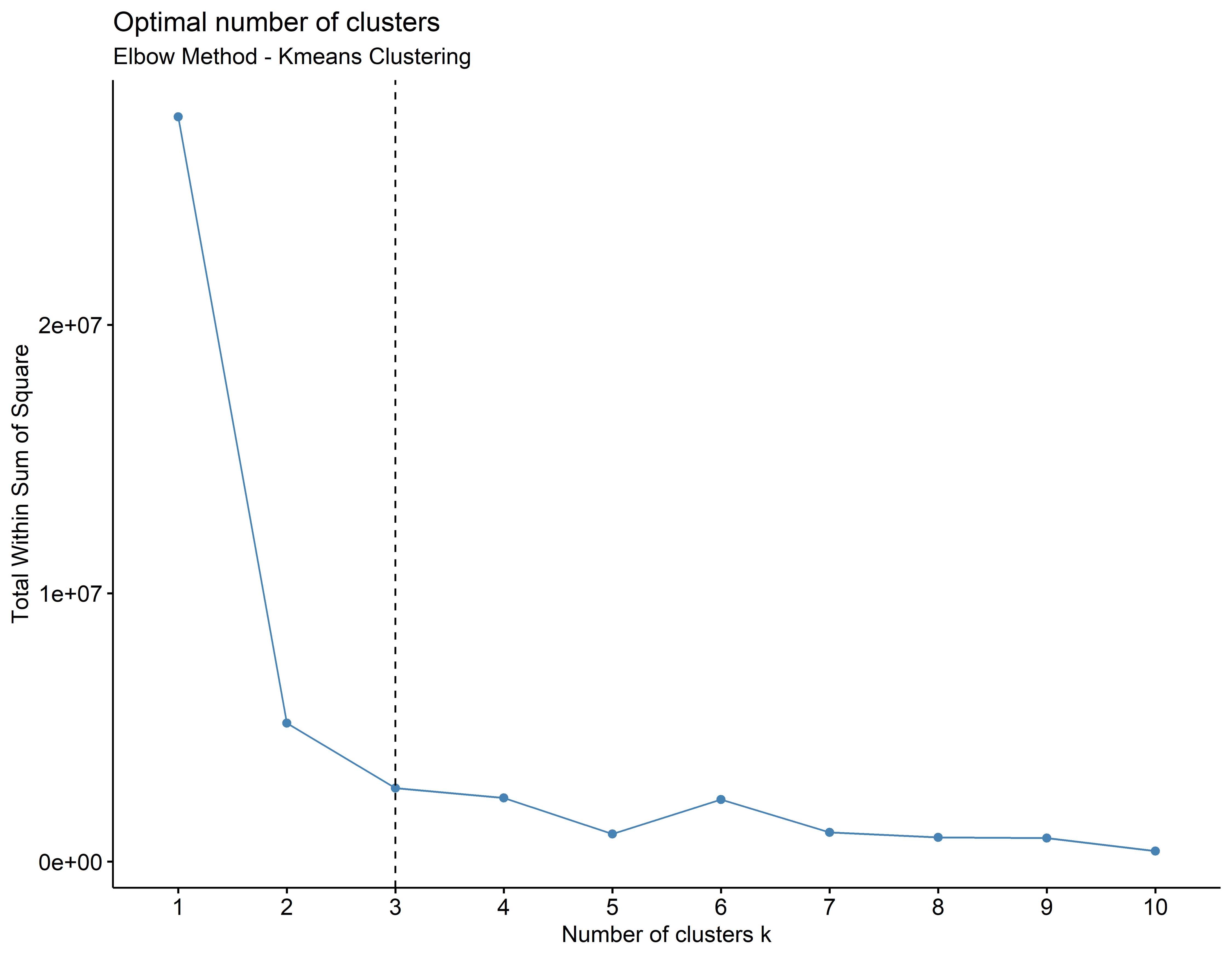

Supplement: S1 Fig — The optimal number of clusters at k = 3 using the wss elbow method. (TIF) [file pcbi.1006937.s001.tif]

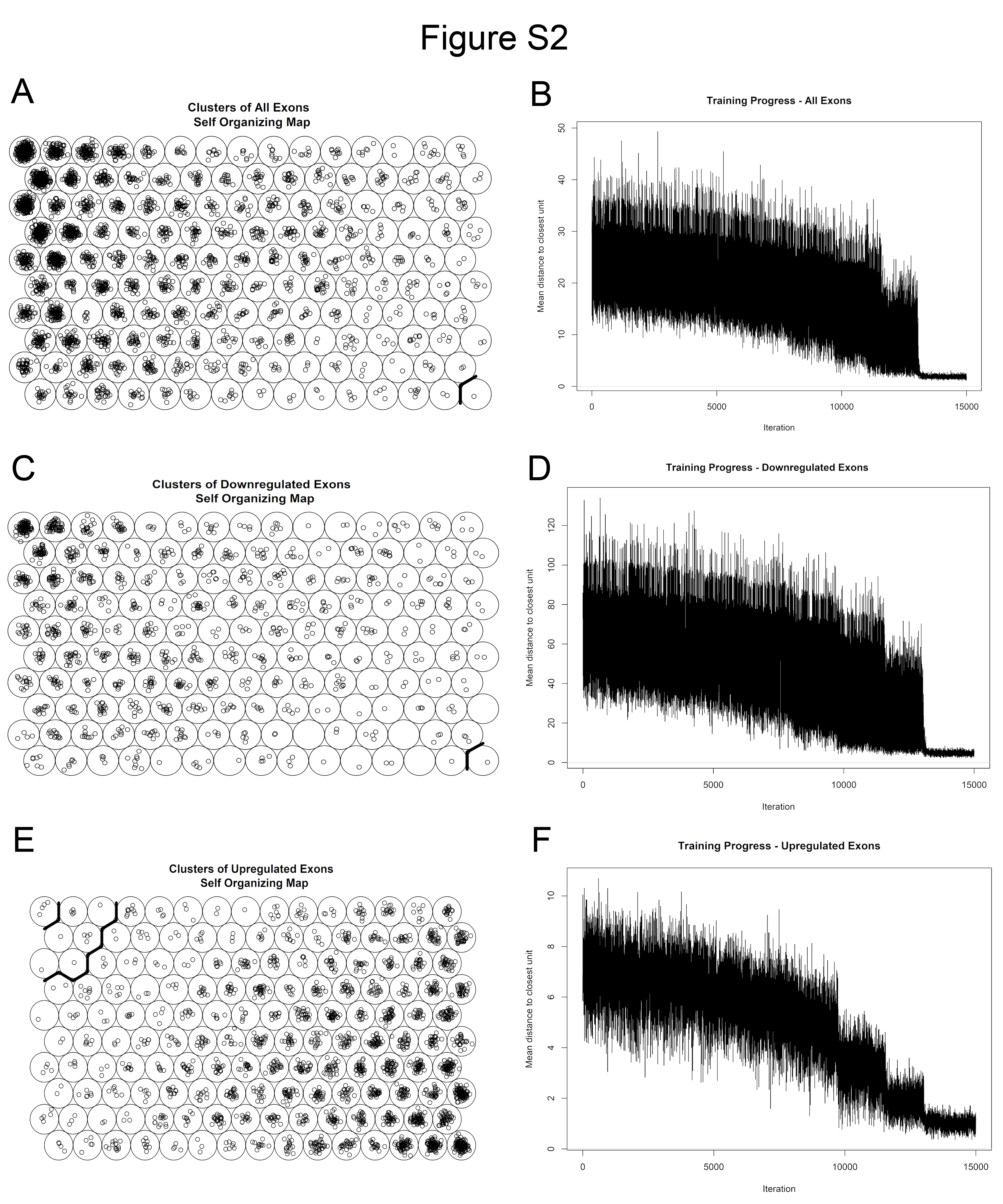

Supplement: S2 Fig — (A) A SOM was applied to the all exons with two partitioned clusters generated by the overlaid hierarchal clustering. (B) The training progress of the SOM model over 15,000 epochs for all exons. (C) A SOM was applied to the down-regulated exons with two partitioned clusters generated by the overlaid hierarchal clustering. (D) The training progress of the SOM model over 15,000 epochs for down-regulated exons. (E) A SOM was applied to up-regulated exons with three partitioned clusters generated by the overlaid hierarchal clustering. (F) The training progress of the SOM model over 15,000 epochs for up-regulated exons. (TIF) [file pcbi.1006937.s002.tif]
