# Supplementary material for: Exon level machine learning analyses elucidate novel candidate miRNA targets in an avian model of fetal alcohol spectrum disorder
Source: PLoS Comput Biol. 2019 Apr 11;15(4):e1006937. doi: 10.1371/journal.pcbi.1006937 (PMC6478348; doi:10.1371/journal.pcbi.1006937)
Supplement: S2 Table — The following table contains the clustered ‘meta-KEGG’ groups that were used to condense various KEGG pathways into clusters for the primary exon analysis. Note that a KEGG pathway can appear in multiple clusters, as dictated by biological function. (DOCX) [file pcbi.1006937.s004.docx]

| **‘Meta’-KEGG Groupings** | **KEGG Pathways in Each Group** |
| --- | --- |
| 1. SNARE interactions in vesicular transport | gga04130: SNARE interactions in vesicular transport  gga03050: Proteasome  gga03060: Protein export |
| 1. FoxO | gga04350: TGF-beta signaling pathway  gga04070: Phosphatidylinositol signaling system  gga04910: Insulin signaling pathway  gga04068: FoxO signaling pathway  gga04310: Wnt signaling pathway  gga04330: Notch signaling pathway |
| 1. Stress response | gga04620: Toll-like receptor signaling pathway  gga03015: mRNA surveillance pathway  gga04060: Cytokine-cytokine receptor interaction  gga04912: GnRH signaling pathway  gga04623: Cytosolic DNA-sensing pathway  gga04142: Lysosome  gga04621: NOD-like receptor signaling pathway  gga04210: Apoptosis  gga04115: p53 signaling pathway  gga04622: RIG-I-like receptor signaling pathway  gga04141: Protein processing in endoplasmic reticulum  gga04145: Phagosome  gga03018: RNA degradation |
| 1. Basal Transcription Factors | gga03020: RNA polymerase  gga03022: Basal transcription factors  gga03040: Spliceosome |
| 1. 1-Carbon Metabolism | gga00670: One carbon pool by folate  gga00790: Folate biosynthesis  gga00730: Thiamine metabolism  gga04122: Sulfur relay system  gga00630: Glyoxylate and dicarboxylate metabolism  gga00480: Glutathione metabolism  gga00750: Vitamin B6 metabolism  gga00920: Sulfur metabolism  gga00240: Pyrimidine metabolism  gga00230: Purine metabolism |
| 1. Cell cycle | gga00030: Pentose phosphate pathway  gga03030: DNA replication  gga00230: Purine metabolism  gga00240: Pyrimidine metabolism  gga04110: Cell cycle |
| 1. Arachidonic acid metabolism | gga00590: Arachidonic acid metabolism |
| 1. DNA repair | gga03030: DNA replication  gga03410: Base excision repair  gga03420: Nucleotide excision repair  gga03430: Mismatch repair  gga03440: Homologous recombination  gga03450: Non-homologous end-joining |
| 1. Isoprene/Chol/Terpene | gga00140: Steroid hormone biosynthesis  gga00120: Primary bile acid biosynthesis  gga00900: Terpenoid backbone biosynthesis  gga00100: Steroid biosynthesis  gga00130: Ubiquinone and other terpenoid-quinone biosynthesis |
| 1. Neural crest development | gga04330: Notch signaling pathway  gga04370: VEGF signaling pathway  gga04916: Melanogenesis  gga04340: Hedgehog signaling pathway  gga04070: Phosphatidylinositol signaling system  gga04012: ErbB signaling pathway  gga04912: GnRH signaling pathway  gga04310: Wnt signaling pathway  gga04210: Apoptosis  gga04115: p53 signaling pathway  gga00830: Retinol metabolism  gga00562: Inositol phosphate metabolism  gga04020: Calcium signaling pathway |
| 1. Endocytosis | gga04144: Endocytosis  gga04145: Phagosome  gga04142: Lysosome  gga04146: Peroxisome |
| 1. mTOR-autophagy-N metabolism | gga04140: Autophagy  gga04150: mTOR signaling pathway |
| 1. Ribosomal Biogenesis | gga03010: Ribosome  gga03020: RNA polymerase  gga03060: Protein export  gga00970: Aminoacyl-tRNA biosynthesis  gga03013: RNA transport  gga04150: mTOR signaling pathway  gga03040: Spliceosome  gga03008: Ribosomal biogenesis in Eukaryotes |
| 1. Cell Adhesion Molecules | gga04514: Cell adhesion molecules (CAMs)  gga04520: Adherens junction  gga04510: Focal adhesion  gga04530: Tight junction  gga04540: Gap junction  gga04810: Regulation of actin cytoskeleton  gga04512: ECM-receptor interaction |
| 1. MAPK signaling pathway | gga04010: MAPK signaling pathway |
| 1. TCA/OxPhos | gga00030: Pentose phosphate pathway  gga00760: Nicotinate and nicotinamide metabolism  gga03320: PPAR signaing pathway  gga00740: Riboflavin metabolism  gga01212: Fatty acid metabolism  gga00730: Thiamine metabolism  gga01210: Oxocarboxylic acid metabolism  gga00640: Propanoate metabolism  gga00770: Pantothenate and CoA biosynthesis |
| 1. Selenocompound metabolism | gga00450: Selenocompound metabolism |
| 1. Cardiomyocyte signaling | gga04260: Cardiac muscle contraction  gga04261: Adrenergic signaling in cardiomyocytes  gga04270: Vascular smooth muscle contraction |
| 1. ABC transporters | gga02010: ABC transporters |
| 1. ErbB signaling pathway | gga04012: ErbB signaling pathway |

**S2 Table. KEGG Groupings.** The following table contains the clustered ‘meta-KEGG’ groups that were used to condense various KEGG pathways into clusters for the primary exon analysis. Note that a KEGG pathway can appear in multiple clusters, as dictated by biological function.
