# Supplementary material for: Exon level machine learning analyses elucidate novel candidate miRNA targets in an avian model of fetal alcohol spectrum disorder
Source: PLoS Comput Biol. 2019 Apr 11;15(4):e1006937. doi: 10.1371/journal.pcbi.1006937 (PMC6478348; doi:10.1371/journal.pcbi.1006937)
Supplement: S3 Table — The unique miRNAs in our dataset and miRNA gene targets identified by the TargetScan7.1 database were placed into DAVID for KEGG enrichment analysis, from which the p-value for each pathway was obtained. Only p-values below 0.05 are reported, except for the focal adhesion pathway under gga-miR-3533-3p and all KEGG pathways of gga-miR-1665. (DOCX) [file pcbi.1006937.s005.docx]

| **miRNA ID** | **KEGG Pathways of TargetScan 7.1 miRNA-Gene Targets** | **P-Value*** |
| --- | --- | --- |
| gga-mir-1647-3p | gga04330:Notch signaling pathway | 0.006 |
| gga-mir-3064-3p | gga04144:Endocytosis  gga04910:Insulin signaling pathway  gga04068:FoxO signaling pathway  gga04931:Insulin resistance  gga04510:Focal adhesion  gga04914:Progesterone-mediated oocyte maturation | 6.94 x 10^-4^  0.002  0.003  0.009  0.009  0.026 |
| gga-mir-3533-3p | gga04150:mTOR signaling pathway  gga04340:Hedgehog signaling pathway  gga04510:Focal adhesion | 0.048  0.052  0.072 |
| gga-mir-6544-3p | gga04510:Focal adhesion  gga04310:Wnt signaling pathway  gga04910:Insulin signaling pathway  gga04012:ErbB signaling pathway  gga04010:MAPK signaling pathway  gga04340:Hedgehog signaling pathway  gga04810:Regulation of actin cytoskeleton  gga04068:FoxO signaling pathway  gga04931:Insulin resistance | 1.77 x 10^-6^  3.49 x 10^-4^  0.001  0.001  0.013  0.018  0.022  0.045  0.046 |
| gga-mir-6590-3p | gga04010:MAPK signaling pathway  gga04120:Ubiquitin mediated proteolysis  gga04914:Progesterone-mediated oocyte maturation  gga03015:mRNA surveillance pathway  gga04810:Regulation of actin cytoskeleton | 0.001  0.012  0.017  0.036  0.051 |
| gga-mir-6593-3p | gga04070:Phosphatidylinositol signaling system  gga04010:MAPK signaling pathway  gga04012:ErbB signaling pathway  gga04320:Dorso-ventral axis formation  gga04910:Insulin signaling pathway  gga04810:Regulation of actin cytoskeleton  gga04510:Focal adhesion | 0.003  0.006  0.006  0.011  0.012  0.038  0.046 |
| gga-mir-6602-5p | gga04070:Phosphatidylinositol signaling system  gga04150:mTOR signaling pathway  gga04510:Focal adhesion  gga04916:Melanogenesis  gga04115:p53 signaling pathway  gga04020:Calcium signaling pathway  gga04310:Wnt signaling pathway  gga03015:mRNA surveillance pathway  gga04810:Regulation of actin cytoskeleton  gga04340:Hedgehog signaling pathway  gga04910:Insulin signaling pathway  gga04931:Insulin resistance  gga04068:FoxO signaling pathway | 5.01 x 10^-4^  7.46 x 10^-4^  0.002  0.006  0.012  0.012  0.012  0.021  0.023  0.027  0.027  0.031  0.034 |
| gga-mir-6604-5p | gga04510:Focal adhesion  gga04020:Calcium signaling pathway | 0.005  0.008 |
| gga-mir-6639-5p | gga04120:Ubiquitin mediated proteolysis  gga04068:FoxO signaling pathway  gga04350:TGF-beta signaling pathway  gga04144:Endocytosis  gga04150:mTOR signaling pathway  gga04810:Regulation of actin cytoskeleton  gga04931:Insulin resistance  gga00534:Glycosaminoglycan biosynthesis - heparan sulfate  gga04012:ErbB signaling pathway  gga04114:Oocyte meiosis  gga04310:Wnt signaling pathway  gga04261:Adrenergic signaling in cardiomyocytes  gga04370:VEGF signaling pathway  gga03015:mRNA surveillance pathway  gga05168:Herpes simplex infection  gga04510:Focal adhesion  gga04070:Phosphatidylinositol signaling system | 3.55 x 10^-4^  6.75 x 10^-4^  0.001  0.002  0.003  0.004  0.006  0.008  0.013  0.016  0.018  0.020  0.027  0.036  0.040  0.041  0.044 |
| gga-mir-6667-5p | gga04510:Focal adhesion  gga04810:Regulation of actin cytoskeleton  gga04910:Insulin signaling pathway  gga04261:Adrenergic signaling in cardiomyocytes  gga04010:MAPK signaling pathway  gga04114:Oocyte meiosis  gga04340:Hedgehog signaling pathway  gga04012:ErbB signaling pathway  gga04912:GnRH signaling pathway  gga04310:Wnt signaling pathway  gga04144:Endocytosis  gga04020:Calcium signaling pathway  gga04931:Insulin resistance  gga05132:Salmonella infection | 8.22 x 10^-4^  0.001  0.002  0.002  0.004  0.007  0.008  0.010  0.010  0.018  0.022  0.024  0.035  0.045 |
| gga-mir-6706-5p | gga04144:Endocytosis  gga04810:Regulation of actin cytoskeleton  gga04910:Insulin signaling pathway  gga04510:Focal adhesion  gga04010:MAPK signaling pathway  gga04012:ErbB signaling pathway  gga04310:Wnt signaling pathway  gga04931:Insulin resistance  gga04114:Oocyte meiosis  gga04150:mTOR signaling pathway | 1.06 x 10^-4^  6.68 x 10^-4^  0.001  0.001  0.003  0.003  0.004  0.006  0.024  0.028 |
| gga-mir-6710-3p | gga04340:Hedgehog signaling pathway  gga04350:TGF-beta signaling pathway  gga04510:Focal adhesion  gga04810:Regulation of actin cytoskeleton  gga04910:Insulin signaling pathway  gga04114:Oocyte meiosis  gga04310:Wnt signaling pathway  gga04068:FoxO signaling pathway  gga04540:Gap junction  gga04931:Insulin resistance | 1.78 x 10^-4^  0.001  0.005  0.008  0.009  0.012  0.029  0.031  0.050  0.051 |
| gga-mir-1650-3p | gga00562:Inositol phosphate metabolism  gga04070:Phosphatidylinositol signaling system  gga04810:Regulation of actin cytoskeleton  gga04010:MAPK signaling pathway  gga04068:FoxO signaling pathway  gga04530:Tight junction  gga04120:Ubiquitin mediated proteolysis  gga04510:Focal adhesion  gga04310:Wnt signaling pathway  gga04144:Endocytosis  gga04350:TGF-beta signaling pathway  gga04910:Insulin signaling pathway | 5.66 x 10^-4^  0.001  0.004  0.008  0.009  0.011  0.011  0.013  0.024  0.037  0.046  0.049 |
| gga-mir-1665-3p | gga04810:Regulation of actin cytoskeleton  gga04115:p53 signaling pathway | 0.068  0.077 |
| gga-mir-6542-3p | gga04010:MAPK signaling pathway  gga04330:Notch signaling pathway  gga04810:Regulation of actin cytoskeleton  gga04068:FoxO signaling pathway  gga04931:Insulin resistance  gga04510:Focal adhesion | 9.52 x 10^-5^  0.004  0.007  0.021  0.045  0.049 |
| gga-mir-6565-3p | gga04931:Insulin resistance  gga04910:Insulin signaling pathway  gga04020:Calcium signaling pathway  gga04510:Focal adhesion  gga04350:TGF-beta signaling pathway  gga04330:Notch signaling pathway  gga04012:ErbB signaling pathway  gga00230:Purine metabolism  gga03020:RNA polymerase | 0.002  0.008  0.016  0.019  0.032  0.034  0.039  0.044  0.047 |
| gga-mir-6565-5p | gga04510:Focal adhesion  gga04010:MAPK signaling pathway  gga04150:mTOR signaling pathway  gga04914:Progesterone-mediated oocyte maturation  gga04012:ErbB signaling pathway  gga04350:TGF-beta signaling pathway  gga00770:Pantothenate and CoA biosynthesis | 0.002  0.002  0.007  0.011  0.013  0.038  0.054 |
| gga-mir-6619-5p | gga04012:ErbB signaling pathway  gga04510:Focal adhesion  gga04010:MAPK signaling pathway  gga04144:Endocytosis  gga04912:GnRH signaling pathway  gga04340:Hedgehog signaling pathway  gga04114:Oocyte meiosis  gga04150:mTOR signaling pathway  gga04540:Gap junction  gga04810:Regulation of actin cytoskeleton  gga04020:Calcium signaling pathway  gga04910:Insulin signaling pathway  gga03015:mRNA surveillance pathway  gga04530:Tight junction  gga04350:TGF-beta signaling pathway  gga04914:Progesterone-mediated oocyte maturation | 1.31 x 10^-4^  1.71 x 10^-4^  4.58 x 10^-4^  0.003  0.007  0.013  0.016  0.021  0.024  0.026  0.027  0.032  0.033  0.045  0.046  0.049 |

**S2 Table.** **Significantly Enriched KEGG Clusters for Each miRNA, as Predicted Using TargetScan.** The unique miRNAs in our dataset and miRNA gene targets identified by the TargetScan7.1 database were placed into DAVID for KEGG enrichment analysis, from which the p-value for each pathway was obtained. Only p-values below 0.05 are reported, except for the focal adhesion pathway under gga-miR-3533-3p and all KEGG pathways of gga-miR-1665.
